# The predictive value of polygenic risk scores for depression in gene-environment interaction studies: a systematic review

**DOI:** 10.1038/s41398-025-03793-7

**Published:** 2026-02-25

**Authors:** Sabrina Illius, Julian Eder, Susanne Vogel, Nina Alexander

**Affiliations:** 1 Department of Psychology, Faculty of Human Sciences, Medical School Hamburg, Am Kaiserkai 1, 20457 Hamburg, Germany; 2 ICAN Institute for Cognitive and Affective Neuroscience, Medical School Hamburg, Am Kaiserkai 1, 20457 Hamburg, Germany; 3 Chair of Biopsychology, Technische Universität Dresden, Zellescher Weg 19, 01062 Dresden, Germany; 4 Department of Psychiatry and Psychotherapy, Philipps University Marburg, Rudolf-Bultmann-Str. 8, 35039 Marburg, Germany; 5 Center for Mind, Brain and Behavior, Philipps University Marburg, Hans-Meerwein-Str. 6, 35032 Marburg, Germany

**Keywords:** Clinical genetics, Predictive markers

## Abstract

**Background:**

According to the diathesis-stress model, genetic liability and environmental exposures interact in the pathogenesis of depression. Polygenic risk scores for depression (PRS_D_) based on large-scale genome-wide association studies have opened new avenues for investigating gene-environment interaction (GxE) beyond candidate gene studies. To the best of our knowledge, this is the first systematic review of studies that have taken a polygenic score approach to study GxE interaction effects on depression phenotypes.

**Methods:**

Based on a preregistered, systematic literature search according to PRISMA guidelines, 56 studies were considered for qualitative analysis. Respective studies investigated a broad range of adverse and protective environmental exposures across the lifespan, e.g., trauma, stressful life events, social environments and (un)healthy lifestyle factors, using cross-sectional and longitudinal designs.

**Results:**

While most studies reported significant main effects of an individual’s PRS_D_ and different environmental exposures on depression phenotypes, the overall evidence for GxE interactions was considerably heterogeneous. Findings of significant PRS_D_xE interactions mostly stem from large cohort studies comprising > 40000 participants, in particular, when recent environmental exposures were considered.

**Conclusion:**

Two general conclusions can be drawn from this review. First, PRS_D_xE interactions, if at all, add a small amount of explained variance in depression phenotypes to the corresponding additive model and may thus require large samples to be reliably detected. Second, in a considerable number of studies, different environmental exposures were found to depend on an individual’s PRS_D_, indicating significant gene-environment correlation. We further discuss limitations, future directions and potential clinical relevance of PRSxE research in depression.

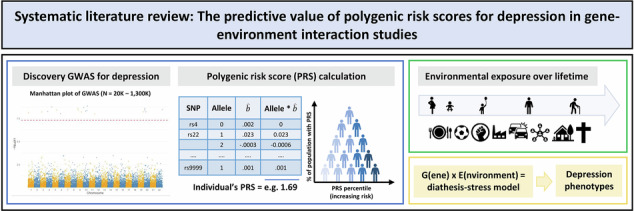

## Introduction

Major depressive disorder (MDD) is one of the most common mental health disorders, with a considerable heritability estimated at approximately 30–40% [1]. The genetic basis of MDD arises from joint effects of many thousands of loci with individual odds ratios of less than 1.2 [2]. According to the diathesis-stress model, genetic liability and environmental exposures interact in the pathogenesis of MDD, thereby explaining why individuals respond so differently to adverse experiences in terms of disease outcome [3]. Early studies investigating gene-environment interactions (GxE) have exclusively focused on candidate genes with known effects on putative biological pathways of MDD. The most prominent finding of a specific GxE interaction relates to the serotonin transporter polymorphism, which has been shown to moderate the influence of life stress on MDD in an influential study [4]. However, meta-analytic evaluations of follow-up studies have seriously questioned the robustness of GxE findings based on single candidate genes [5]. Within the last decade, large-scale genome-wide association studies (GWAS) on a broad spectrum of depression phenotypes have now opened new avenues for GxE research. In 2019, a meta-analysis drawing on 807553 individuals from the three largest GWAS of depression at that time [6–8] identified 102 independent variants associated with depression, of which 87 replicated in an independent sample [9]. Recently, an even larger meta-analysis (n > 1 million participants) yielded 233 genetic variants associated with depression [10]. Analogously, first genome-wide by environment interaction studies (GWEIS) of depression and stressful life events (SLE) have already successfully identified GxE interactions in a hypothesis-free approach (e.g., [11–13]). However, GWEIS require overly large sample sizes to achieve adequate statistical power which hampers an in-depth assessment of environmental exposures and (intermediate) phenotypes of depression. To this end, polygenic risk scores (PRSs) have been advocated as a promising strategy to integrate genetic liability into a single measure, thereby allowing a hypothesis-free study of GxE effects with higher statistical power as compared to GWEIS. PRSs are commonly calculated based on a set of top-ranking genetic markers identified by prior GWAS and represent the weighted sum of their trait-associated alleles [14]. The predictive power of polygenic risk scores for depression (PRS_D_) has steadily increased in parallel with rising GWAS discovery sample sizes [15]. While early GWAS from the Psychiatric Genomics Consortium (PGC) explained only around 0.5% of the variance in depression liability (n = 18759) [16], PRS_D_ derived from more recent large-scale GWAS now explain approximately 1.5–3.2% (n = 807553) [9]. Consequently, PRS_D_ are increasingly recognized as a promising approach to test the diathesis–stress model of depression [3]. For example, recent studies have now begun to address PRS_D_ as a potential moderator of well-established environmental risk factors of depression, including different types of childhood trauma (CT [17–31]) and other forms of early [31–38] and prenatal [39–43] adversity, as well as adult trauma/stressful life events (SLE) [23, 28, 31, 44–52], social environments [53–57], (un)healthy lifestyle factors (e.g., [58–63]) and cumulative environmental adversity [64–70]. Such GxE studies searched for departures of PRS_D_ and environmental exposure effects from additivity (i.e., combined effects differ from their sum) or multiplicativity (i.e., combined effects differ from the product). At the same time, recent GxE studies leveraging PRS_D_ face several methodological challenges, including heterogeneity in the measurement of environmental exposures and depression outcomes, lack of consensus on the optimal threshold for PRS_D_ construction, and potential confounding. A key source of such confounding is gene–environment correlation (rGE), the non-random association between genetic predisposition and environmental exposures, which can arise through passive, evocative, or active mechanisms [71, 72]. For example, individuals with higher genetic liability for depression may be more likely to encounter environmental adversity, thereby mimicking or inflating GxE interaction effects [27, 73].

In light of both promises and challenges of this field of research, the aim of this systematic review was to summarize and critically discuss the rapidly growing number of studies using PRS_D_ to investigate GxE interaction effects on depression-related outcomes. Given the substantial methodological and conceptual heterogeneity outlined above, we opted for a narrative rather than a quantitative, meta-analytic synthesis.

## Material and methods

### Search strategy

The systematic literature search was based on PRISMA guidelines [74] using Excel and Zotero and conducted up to May 15th, 2024 using PubMed, Embase (Ovid), MEDLINE (Ovid), Scopus, Web of Science Core Collection, Medline – Web of Science and PsycINFO. The following search terms were used: (Depression OR “Depressive Disorder” OR “Major Depression”) AND (“Polygenic Score” OR “Polygenic Risk”). All peer-reviewed journal articles released up to this date were considered. Two authors independently reviewed all identified journal articles (SI, JE). Where conflicts occurred a third author was consulted (NA).

### Inclusion and exclusion criteria

We considered all English language original studies published until May 15th, 2024, that investigated interactions of PRS_D_ and environmental exposures (both risky and protective) on depression-related phenotypes and behaviors in healthy and clinical human samples of any ethnic origin and age. Depression-related phenotypes included intermediate phenotypes such as neural, endocrine and immunological changes associated with major depression (MD). Conference abstracts, published abstracts, preprints, editorials, reviews as well as studies that had used PRS_D_ to predict GxE interactions on other mental diseases (e.g., schizophrenia) were not considered in this review. We refined this data set by selecting studies that met the following inclusion criteria: i) PRS_D_ calculation based on whole-genome summary statistics of GWAS using definitions from the spectrum of depressive phenotypes like lifetime MDD, depressive symptoms or alternative methods to measure MD, ii) no overlap between GWAS discovery sample used for PRS_D_ calculation and target samples of the GxE study for the same phenotype; studies with overlapping samples were only included if outcomes differed (e.g., biomarkers), iii) analysis of GxE interactions. The review was registered to PROSPERO in advance (registration number CRD42021276066).

### Quality assessment

While no validated quality or risk-of-bias assessment tool exists for PRS studies, particularly those investigating GxE interactions, we considered it important to conduct a structured narrative quality assessment to provide at least some evaluation of methodological rigor. We adapted the methodological approach developed by Sharew et al. [75] for systematic reviews of PRS studies, comprising seven core criteria (Q1–Q7) covering: power calculation, clarity of in-/exclusion criteria, presence of an external validation cohort, adjustment for multiple testing, specification of PRS construction methods, type of association analysis, and consideration of covariates/confounders. To capture additional study characteristics relevant to quality, bias, and interpretability, we also extracted information on study design, study sample size, GWAS discovery sample size, assessment of ethnicity, *p*-value threshold (pT) used for PRS calculation, method of environmental exposure and phenotype assessment, and whether rGE was assessed. The quality assessment was performed by SI under the supervision of NA. Full definitions of Q1–Q7 and the coding schemes for these additional variables are provided in Supplementary Tables 2 and 3.

### Data extraction

A data extraction form was developed through discussion between all authors. Items were included based on expert knowledge and relevance to the review topic. The form was piloted with two publications by each reviewer independently and updated before being applied to all studies. Extracted data were checked by a second reviewer. For each study, we extracted information about authors, year of publication, study design, sample characteristics (size, ancestry, age, sex), PRS_D_ calculation (GWAS that provided the basis for PRS_D_ calculation, *p*-value threshold), type of environmental exposure, predicted depression-related outcome and main study results, including GxE interaction effects, main effects and gene-environment correlations (rGE, i.e., whereby an individual’s PRS_D_ influences the exposure to specific environments), if reported. For the classification of results as significant or non-significant, we adopted the significance thresholds reported by the original studies. Reported effect size estimates (e.g., odds ratios (OR), hazard ratios (HR), incidence rate ratios (IRR), relative excess risk due to interaction (RERI), standardized regression coefficients (*β*), unstandardized regression coefficients (b), correlation coefficient (r)) and explained variance (R²) were extracted whenever available. If multiple models or effect estimates were reported within a study, we extracted the largest effect size. This could reflect either the strongest positive association for risk effects (e.g., positive β or OR/HR > 1) or the strongest negative association for protective effects (e.g., most negative β or OR/HR < 1). Only information provided in original articles and supplements was included.

## Results

### Study selection and characteristics

**Fig. 1:**
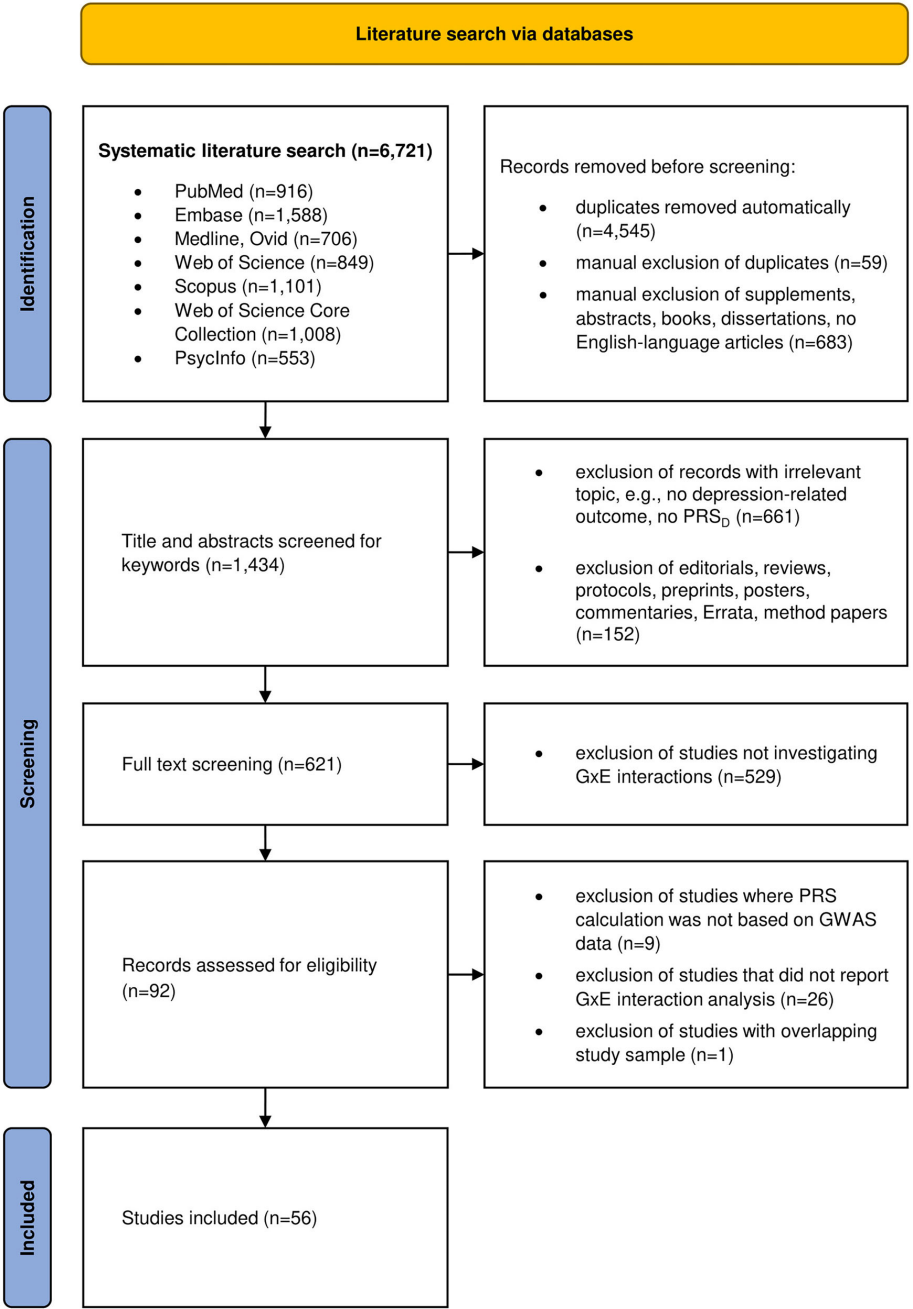
PRISMA flow chart of the systematic literature search.

A detailed flow chart of the systematic literature search is depicted in Fig. 1. In total, the literature search identified 6721 records. After exclusion of duplicates, supplements, conference abstracts, books, dissertations and non-English language articles, 1434 records remained. In a next step, abstracts were screened to remove reviews, editorials and records with irrelevant topics. Remaining records (n = 621) were screened in full-text and those studies not investigating GxE interactions were excluded. This resulted in 92 records assessed for eligibility. Out of these studies, those using PRS_D_ that were not based on GWAS data or those not reporting GxE interaction analyses were excluded, resulting in a final number of n = 56 studies for qualitative analysis.

Study results were grouped into those addressing interactions between an individual’s PRS_D_ and i) early environmental exposures (25/56 studies, 44.6%) and ii) adult/cumulative environmental exposures (26 of 56 studies, 46.4%) on depression-related outcomes, with n = 5 studies reporting data on both (8.9%). Studies on early exposures focused on different types of CT, but also contributed data on other forms of adversity, e.g., adoption, peer victimization, socioeconomic status (SES), urbanization, and prenatal adversity. The majority of studies on adult exposures investigated different types of trauma or SLEs, whereas others addressed an individual’s social environment as well as other protective (e.g. healthy lifestyle) and risk (e.g. urbanicity) factors. In terms of depression-related outcomes, across the 56 studies a total of 88 distinct outcomes were reported. Most of these referred to depression risk or depressive symptoms (70/88, 79.5%), whereas others focused on intermediate phenotypes such as brain structure and functioning or low-grade inflammation (18/88, 20.5%). PRS_D_ calculations in individual studies were based on GWAS on depressive phenotypes, including lifetime MDD, depressive symptoms or alternative methods to measure MD [6, 7, 9, 10, 16, 76–81] (Supplementary Table 1). The study samples cover a broad age range from infancy to late adulthood.

### Quality assessment results

Most studies (47/56; 83.9%) were conducted in samples of European ancestry, while nine studies (16.1%) included participants from other ancestries. With one exception, all discovery GWAS used for PRS_D_ calculation were based on European ancestry populations. Target sample sizes ranged from 105 to 490780 participants (median = 3428), and discovery GWAS sample sizes ranged from 15298–1306090. Studies employed cross-sectional (exposure and outcome assessed at the same time point in the same sample), retrospective longitudinal (exposure measured before the outcome in time, but both obtained from past records and analyzed after outcome occurrence), or prospective longitudinal designs (both environmental exposures and depressive outcomes were prospectively assessed).

All studies described inclusion and exclusion criteria. Only a minority (8/56; 14.3%) reported a formal power calculation, and all studies considered relevant covariates or confounders in their analyses. Correction for multiple testing was applied in 33/56 studies (58.9%), while external cohort validation was performed in only four studies (7.1%). More than half of the studies (31/56; 55.4%) examined rGE. In terms of methodological specification, all studies detailed their PRS_D_ construction approach, with considerable variability in the *p*-value thresholds applied. The type of analysis used for the reported results was always specified. Environmental and phenotype assessments included objective measurement, established interview, register-based assessment, established self-report questionnaire, self-constructed measurement, and biomarker-based assessment. A detailed overview of all quality assessment ratings (Q1–Q7 and additional coded variables) is provided in Supplementary Table 4, with definitions in Supplementary Tables 2 and 3. In the absence of a validated quality or risk-of-bias tool for PRS-based GxE studies, these ratings should be interpreted with caution, as the assessment may not encompass all bias-relevant aspects important to this field.

### Studies investigating the interaction between PRS_D_ and early environmental exposures on depression-related outcomes

#### Childhood trauma

Most studies on early exposures investigated the interaction of PRS_D_ and different types of CT (Table 1), which are among the strongest risk factors for developing MDD [25]. In a first cross-sectional study on 1645 adult MDD patients and 340 controls from the Netherlands Study of Depression and Anxiety (NESDA), Peyrot et al. [26] identified a significant additive and multiplicative interaction of PRS_D_ and CT (different types of abuse) that explained 0.5% of the variance in MDD risk. Specifically, the effect of PRS_D_ in predicting MDD and severe (chronic or recurrent) MDD risk was found to be increased in the presence of CT as assessed by a DSM-based interview. In an attempt to replicate these findings, Mullins et al. [23] reported a significant, but opposing multiplicative interaction in 240 MDD adult patients and 272 controls of the RADIANT UK cohort, explaining 1.9% of the variance in MDD. In this cross-sectional study, a higher PRS_D_ was associated with an increased MDD risk in those individuals unexposed to CT (neglect and abuse). In order to resolve these inconsistencies, Peyrot et al. [27] reanalyzed data from both cohorts with an updated, more accurate PRS_D_ [7] and meta-analyzed their results together with seven additional cohorts. Drawing on a total of 3024 MDD patients and 2741 controls, this study revealed no meta-analytic evidence for a significant interaction between PRS_D_ and self-reported CT on MDD risk in adulthood, irrespective of the specific CT domain. Comparable findings stem from a study on 1359 Syrian refugees, where the associations of war events and self-reported internalizing symptoms were found to be unmoderated by the child’s PRS_D_ [29]. Likewise, another cross-sectional analysis of 5853 children from the Adolescent Brain and Cognitive Development (ABCD) study found no interaction between PRS_D_ and a composite score of parent-reported CT (and other life stressors) on suicidal ideation or attempts [21]. Only recently, however, significant PRS_D_ x CT interaction effects on interview-derived depressive symptoms were reported in 38945 participants of the Lifelines Cohort Study (Lifelines), explaining up to 0.09% of variance [31]. Here, different types of neglect and abuse (except sexual abuse) were found to amplify the effect of an individual’s PRS_D_ across multiple *p*-value thresholds. In contrast to the Lifelines findings, a similarly large study of 35633 UK Biobank (UKB) participants found no evidence for a PRS_D_ x CT interaction, although both PRS_D_ and CT showed significant main effects on depression risk [17].

**Table 1:** Studies investigating the interaction between a PRS_D_ and early environmental exposures on depression-related outcomes.

study	study design^1^	population (ancestry)	age (mean)	female (%)	PRS discovery sample	PRS p-value threshold (pT)	environmental exposure	predicted outcome	main results (effect size estimate [CI])
*Prenatal adversity*									
Chen et al., [43]	prospective longitudinal	cohort 1: (European) 5546	t1 = 4 yrs (6 follow-ups until age 16)	49.1	MTAG-2018	range: p= 0.01–1.00 at intervals of 0.01	prenatal maternal depression (EPDS) and anxiety (CCEI)	internalizing symptoms (SDQ)	• no sign. multiplicative interaction of PRS_D_ and prenatal maternal depression (β = 0.004 [–0.031, 0.039]) and anxiety (β = 0.036 [0.002, 0.071]) on internalizing symptoms • sign. main effects of PRS_D_ (β = 0.035 [0.015, 0.055]), prenatal maternal depression (β = 0.079 [0.052, 0.106]) and anxiety (β = 0.062 [0.036, 0.089]) • sign. correlation of PRS_D_ and prenatal maternal depression (r = 0.061) and anxiety (r = 0.069)
		cohort 2: (European) 514	t1 = 3.4 yrs t2 = 8.7 yrs				prenatal maternal depression (CES-D)	internalizing symptoms (CBCL)	
Wang et al., [42]	prospective longitudinal	2938 mother-child dyads (European)	t1 = 10.6 yrs t2 = 13.8 yrs	49.8	PGC-MDD-2018 (excluding 23andMe)	<0.05	prenatal maternal vitamin D status	depression (SMFQ)	• no sign. interaction of PRS_D_ and prenatal maternal vitamin D status on childhood or adolescent depression (largest OR = 4.09 [1.26, 13.30]) • sign. main effect of PRS_D_ for childhood depression (largest OR = 1.96 [1.24, 3.03]), no sign. main effect of vitamin D status (largest OR = 1.32 [0.98, 1.79])
*Childhood trauma and aversive experiences*									
Chen et al., [17]	cross-sectional	35633 (European)	55.7 yrs	62.6	UKB-2020	<5 x 10^-8^	adverse exposures (TFMHQ)	depression age of onset (self-report)	• no sign. GxE interaction with CT (β = −0.550) • sign. main effects of PRS_D_ (β = –1.601) and adverse exposures (felt hated: β = –7.385)
Halldorsdottir et al., [19]	cross-sectional	cohort 1 (European): 279 MDD 187 HC	14.8 yrs 14.7 yrs	68.0 63.0	PGC-MDD-2018	7 pTs tested (range: <5 x 10^-8^ to <0.10; final pT: <0.05)^2^	CT (LES, MEL)	depression (DISC, DIJK, BDI-II)	• no sign. multiplicative interaction of PRS_D_ and CT on MDD status (OR = 0.838 [0.502, 1.401]), depression severity (β = –0.187 [–0.444, 0.069]), age of onset (β = –0.321 [–0.934, 0.292]) • sign. main effect of PRS_D_ on case-control status (OR = 1.560 [1.230, 1.980], R² = 7.9%), depression severity (β = 0.177 [0.021, 0.288], R² = 7.7%), and age at onset (β =−0.375 [−0.688, −0.062], R² = 45.9%), main effect of CT NR
	prospective longitudinal	cohort 2 (European): 756 depressed 694 non-depressed	t1 = 14.0 yrs t2 ≈ 14.6 yrs t3 ≈ 15.0 yrs t4 ≈ 16.0 yrs	63.0			CT (CTQ)	depression(CDI)	• no sign. multiplicative interaction of PRS_D_ and CT on (prospective) MDD status (HR = 0.877 [0.592, 1.300]), depressive symptoms (β = 1.010 [–0.301, 2.320]) • sign. main effect of PRS_D_ on (prospective) MDD status (HR = 1.202 [1.045, 1.383], R² = 4.4%), depressive symptoms (β = 0.557 [0.167, 0.947], R² = 7.5%), main effect of CT (NR)
Iob et al., [20]	retrospective longitudinal	3428 (British)	t1 ≈ 56.7 yrs (8 follow-ups over 14 yrs)	55.4	PGC-MDD & 23andMe 2019	=1.0	CT (ELSA’s Life History interview), parental bonding (PBI)	depressive symptoms (CESD-8)	• sign. additive and multiplicative interaction of PRS_D_ and CT on depressive symptom trajectories (largest OR = 1.13 [1.04, 1.23]) • sign. additive and multiplicative interaction of PRS_D_ and low parental bonding on depressive symptom trajectories (largest OR = 1.47 [1.15, 1.89]) • sign. main effects of PRS_D_ (largest OR = 1.47 [1.28, 1.70]), CT (largest OR = 1.44 [1.30,1.60]) and low parental bonding (largest OR = 2.36 [1.70, 3.28])
Joo et al., [21]	cross-sectional	5853 (multi-ethnic)	9.9 yrs	52.8	MDD-2019	=1.0	early life stress (FES & KSADS-5, PTSD module)	suicidal ideation, suicide attempt (KSADS-Comp)	• no sign. interaction of PRS_D_ and early life stress on suicidal ideation (NR) or suicide attempts (NR) • sign. main effect of PRS_D_ in a subsample of European ancestry (largest OR = 1.12 [1.04, 1.21], Pseudo R² = 2.9%), main effect of early life stress NR
Mullins et al., [23]	cross-sectional	240 MDD 272 HC (European)	44.7 yrs NR	73.5 NR	PGC-MDD-2013 (excluding RADIANT UK data)	9 pTs tested (range: <0.0001 to <0.5)	CT (CTQ)	depression (SCAN)	• sign. multiplicative interaction of PRS_D_ and CT on MDD status (threshold specific, largest R² = 1.9%), no sign. additive interaction (largest OR = 1.00 [0.99, 1.00]) • no sign. main effect of PRS_D_ (OR = 1.18 [0.98, 1.42], Pseudo R² = 0.7%), sign. main effect of CT (R² = 30.2%) • no sign. correlation of PRS_D_ and CT (largest OR = 1.76 [0.49, 6.28])
Musci et al., [24]	retrospective longitudinal	488 (mostly African American)	t1 ≈ 11.9 yrs t2 ≈ 12.9 yrs t3 ≈ 13.9 yrs t4 ≈ 14.9 yrs t5 ≈ 15.9 yrs t6 ≈ 16.9 yrs t7 = 17.8 yrs	45.6	PGC-MDD-2013	<0.05	parental death and/or divorce by age 6 (HDLF)	internalizing symptoms (BHIF-A)	• no sign. interaction of PRS_D_ and parental death/divorce on internalizing symptoms (β = 0.011) • sign. main effects of PRS_D_ (β = 0.109 [−0.141, 0.085]) and parental death/divorce (occasion-specific; largest β = −0.352 [−0.573, −0.005])
Peyrot et al., [26]^3^	cross-sectional	1645 MDD 340 HC (European)	42.2 yrs 43.3 yrs	68.0 57.0	PGC-MDD-2013 (excluding NESDA data)	8 pTs tested (range: <0.001 to <0.5)	CT (CTI)	lifetime MDD (CIDI)	• sign. additive (RERI = 0.37 [0.14, 0.71]) and multiplicative (largest OR = 1.16) interaction of PRS_D_ and CT (for emotional neglect and physical abuse) on MDD risk • sign. main effects of PRS_D_ (largest OR = 1.22) and CT (OR = 1.69) • no sign. correlation between PRSD and CT (largest β = 0.02)
Peyrot et al., [27]	meta-analysis on mixed design studies	9 PGC cohorts (European): 3024 MDD 2741 HC	36.3-53.6 yrs	50.0- 70.0	PGC-MDD-2018 (excluding data from cohorts of this study)	5 pTs tested (range: <0.01 to <1)	CT (CTQ and other self-report measures)	MDD (DSM-criteria based diagnostic interviews)	• no sign. additive (largest RERI = 0.84 [−0,52, 22.18]) or multiplicative (largest OR = 1.05 [0.91, 1.20]) interaction of PRS_D_ and CT on MDD risk. • sign. main effects of PRS_D_ (largest OR = 1.35 [1.22, 1.48], R² = 1.70%) and CT (largest OR = 2.63) • sign. correlation of PRS_D_ and CT (β = 0.76)
Schür et al., [28]	retrospective longitudinal	516 soldiers (European)	t1 = 29.6 yrs t2 ≈ 29.7 yrs t3 ≈ 30.2 yrs t4 ≈ 31.2 yrs t5 ≈ 32.2 yrs	8.5	PGC-MDD-2018	13 pTs tested (range: <5x10^-8^ to <1)	CT (ETI)	depressive symptoms (SCL-90)	• no sign. interaction of PRS_D_ and CT on baseline (largest β = −0.332) and post-deployment depressive symptoms (largest β = –1.035) • no sign. main effect of PRS_D_ (largest β = 1.154), main effect of CT NR • nominally sign. correlation of PRS_D_ and CT (β = 0.09)
Smeeth et al., [29]	cross-sectional	1359 Syrian refugees (Middle Eastern ancestry)	11.3 yrs	52.8	UKB-portability-2022	NR	war events (WEQ)	depressive symptoms (CES-DC) trauma symptoms (CPSS)	• no sign. interaction of PRS_D_ and war events on composite score of internalizing symptoms (OR = 0.86 [0.68, 1.09]) • no sign. main effect of PRS_D_ (OR = 0.67 [0.24, 1.88]), sign. main effect of war events (OR = 0.93 [0.90, 0.95])
Taylor et al. [30]	cross-sectional	2050 (British)	75.2 yrs	65.8	PGC-MDD & 23andMe 2019	=1.0	CT (ELSA’s Life History interview)	depressive symptoms (CESD-8)	• no sign. interaction of CT and PRS_D_ on depressive symptoms during the Covid-19 pandemic (OR = 0.944 [0.849, 1.049]) • no sign. main effects of PRS_D_ (OR = 1.135 [0.934, 1.386])_,_ sign. main effect of CT (OR = 1.250 [1.124, 1.386])
Wang et al., [31]	cross-sectional	38945 (European)	44.1 yrs	59.6	PGC-MDD & 23andMe 2019	11 pTs tested (range: <5 x 10^-8^ to <1)	CT (CTQ)	depressive symptoms (MINI)	• sign. interaction of PRS_D_ and CT on depressive symptoms (largest β = 0.0384, R² = 0.09%) • sign. main effects of PRS_D_ (largest β = 0.1149, R² = 0.69%) and CT (β = 0.3191, R² = 5.91%) • sign. correlation of PRS_D_ and CT (r = 0.08)
*Other forms of childhood adversity and protective factors*									
Agerbo et al., [32]	retrospective longitudinal	17098 MDD 18582 HC (Danish)	t1 = 10 yrs (continuous follow-up up to 21 yrs)	68.7 49.2	MDD-2019 (excluding iPSYCH)	10 pTs tested (range: <5x10^-8^ to <1)	childhood SES (register-based)	early-onset depression (clinical records)	• no sign. interaction of PRS_D_ and SES (t1) on early-onset depression (NR) • sign. main effects of PRS_D_ (HR=1.32 [1.29, 1.35]) and SES (largest HR = 1.65 [1.45, 1.87]) • sign. correlation of PRS_D_ and childhood SES (largest r = –0.058)
Armitage et al., [33]	prospective longitudinal	2268 (European)	t1 = 13.0 yrs t2 = 23.0 yrs	63.9	PGC-MDD & 23andMe 2019	11 pTs tested (range: <5x10^-8^ to <0.5)	peer victimization (BFIS)	depressive symptoms (SMFQ), well-being (WEMWBS)	• no sign. interaction of PRS_D_ and peer victimization on prospective depressive symptom development (largest β = 0.024 [–0.022, 0.070]) • no sign. interaction of PRS_D_ and peer victimization on well-being (largest β = –0.085 [–0.554, 0.384]) • sign. main effects of PRS_D_ (largest β = 0.100 [0.017, 0.184]) and peer victimization (largest β = 0.188 [0.084, 0.293]) on prospective depressive symptoms • no sign. main effects of PRS_D_ (largest β = 0.091 [–0.702, 0.885]) and peer victimization (largest β = –0.014 [–0.979, 1.010]) on well-being • sign. correlation of PRS_D_ and peer victimization (largest β = 0.163 [0.059, 0.266], R² = 0.42% at pT = 0.3)
Chen et al., [17]	cross-sectional	35633 (European)	55.7 yrs	62.6	UKB-2020	<5x10^-8^	protective exposures (TFMHQ)	depression age of onset (self-report)	• sign. interaction of PRS_D_ and felt loved as a child on depression age of onset (β = 0.708), effect driven by males (β = 1.421) • sign. main effects of PRS_D_ (β = –1.601) and adverse/protective exposures (felt loved: β = 5.534)
Kosciuszko et al., [34]	retrospective longitudinal	6202 (British)	65.2 yrs (5 follow-ups over 14 yrs)	52.2	PIR-2021	NR	SES (self-report educational attainment)	depressive symptoms (CES-D)	• sign. interaction of PRS_D_ and educational attainment on baseline depressive symptoms (β = −0.01 [−0.02, −0.01]), but not on rate of change in depressive symptoms (β = −0.00 [00.00, 0.00]) • sign. main effect of PRS_D_ (β = 0.35 [0.27, 0.44]) and educational attainment (β = −0.06 [−0.07, −0.05]) on baseline depressive symptoms • no sign. main effects of PRS_D_ (β = 0.02 [−0.04, 0.01]) and educational attainment (β = 0.001 [−0.001, 0.004]) on rate of change in depressive symptoms
Lehto et al., [35]	cross-sectional	3151 adopted 240329 non-adopted (European)	56.9 yrs	54.4	SSGAC-2016 (excluding UK Biobank data)	7 pTs tested (range: <5x10^-8^ to <0.5; final pT: <0.5)^3^	adoption (self-report)	depressive symptoms (self-report)	• no sign. interaction of PRS_D_ and adoption on depressive symptoms (β = −0.01 [−0.05, 0.03], R² = 1.97%) • sign. main effects of PRS_D_ (β = 0.05 [0.05, 0.06], R²= 1.93%) and adoption (β = 0.10 [0.05, 0.14], R²= 1.66%) • sign. correlation of PRS_D_ and adoption (OR = 1.066 [1.03–1.11])
Misztal et al., [82]	cross-sectional, prospective longitudinal	4975 (European)	9-10 yrs (followed-up longitudinally)	47.0	PGC-MDD & 23andMe 2019	<0.05	sport frequency and sport type (parent report)	anxious/ depressed, withdrawn/ depressed symptoms (CBCL)	• no sign. interaction of PRS_D_ and sport type/frequency on anxious/depressed and withdrawn/depressed symptoms (NR) • sign. main effects of PRS_D_ (NR) and sport type/frequency (e.g., team sports: β = −0.09 [−0.11, −0.06])
Nelemans et al., [36]	prospective longitudinal	327 mother-child dyads with low SES (European)	t1=13.0 yrs t2=14.0 yrs t3=15.0 yrs t4=16.0 yrs t5=17.0 yrs t6=18.0 yrs	43.4	PGC- 23andMe-2016	<5x10^-8^; sensitivity analysis: 12 pTs tested (range: <5x10^-7^ to <0.5)	critical parenting t1-t6 (LEE, multi-informant)	depression (RADS-2)	• sign. interaction of PRS_D_ (threshold specific) and critical parenting on intercept levels of adolescent depressive symptoms (largest β = 0.15) and changes over time (largest β = 0.25) • sign. main effect of PRS_D_ (different thresholds) on mean depressive symptoms (largest r = 0.20), but not on changes over time (NR), sign. main effect of critical parenting (largest r = 0.43) • sign. correlation of PRS_D_ and critical parenting (largest r = 0.21)
Perret et al., [37]	prospective longitudinal	748 (Canadian)	t1≈12-13 yrs t2≈15-17 yrs	55.5	MDD-2019	4 pTs tested: (<0.01; <0.10; <0.50; <1.00)	peer victimization (multi-informant: SRVS, Behavior Questionnaire)	depressive symptoms (MAI)	• no sign. interaction of PRS_D_ and peer victimization (t1) on depressive symptoms (t2) (largest β = 0.039) • sign. main effects of PRS_D_ (largest β = 0.10) and peer victimization (largest β = 0.34) • sign. correlation of PRS_D_ and self-reported (but not teacher reported) peer victimization (r = 0.11)
Wang et al., [31]	cross-sectional	overall sample: 38945 (European)	44.1 yrs	59.6	PGC-MDD & 23andMe 2019	11 pTs tested (range: <5x10^-8^ to <1)	long-term difficulties; SLE; social support (parent-report)	depressive symptoms (MINI)	overall sample: • sign. interaction of PRS_D_ and long-term difficulties (largest β = 0.0541, R² = 0.17%), SLE (largest β = 0.0385, R² = 0.05%) and social support (largest β = –0.0417, R² = 0.09%) on depressive symptoms • sign. main effects of PRS_D_ (β = 0.1105, R² = 0.66%), long-term difficulties, SLE and social support (largest β = 0.4195, R² = 9.54%) • sign. correlation of PRS_D_ and long-term difficulties, SLEs, social support (largest r = 0.08)
		child subsample: 2865 (European)	12.4 yrs	50.3				depressive symptoms (CBCL, YSR)	child subsample: • no sign. interaction of PRS_D_ and long-term difficulties (largest β = 0.0862, R² = 0.15%), SLE (largest β = 0.0352, R² = 0.12%) and social support (largest β = 0.0229, R² = 0.12%) on depressive symptoms • sign. main effects of PRS_D_ (in children: β = 0.0403, R² = 0.21%), long-term difficulties, SLE and social support (largest β = 0.6235, R² = 7.59%)

*b* unstandardized regression coefficient, *BDI-II* Becks-Depression-Inventar Revision, *BFIS* Bullying and Friendship Interview Schedule, *BHIF-A* Baltimore How I Feel questionnaire – Adolescent Report, *CBCL* Child Behavior Checklist, *CCEI* Crown Crisp Experiential Index, *CDI* Children’s Depression Inventory, *CES-D* Center for Epidemiologic Studies Depression Scale, *CESD-8* 8-item version of the Centre for Epidemiological Studies Depression, *CES-DC* Center for Epidemiological Studies Depression Scale for Children, *CI* confidence interval, *CIDI* Composite International Diagnostic Interview for DSM-IV, *CPSS* Trauma Symptoms Checklist, *CT* childhood trauma, *CTI* Childhood Trauma Interview, *CTQ* Childhood Trauma Questionnaire, *DIJK* Depressions Inventar für Kinder und Jugendliche, *DISC* Depression Intensity Scale Circles, *DSM* Diagnostic and Statistical Manual of Mental Disorders, *EPDS* Edinburgh Postnatal Depression Scale, *ELSA* ELSA ’s (English Longitudinal Study of Ageing) Life History interview, *ETI* Early Trauma Inventory, *FES* Youth Family Environmental Scale, *HC* healthy controls, *HDLF* Life Change Events subscale from the Health and Daily Living Form, *HR* hazard ratio, *KSADS(-Comp)* Kiddie Schedule for Affective Disorders and Schizophrenia, *LEE* Level of Expressed Emotions Scale, *LES* Life Event Survey, *MAI* Mental Health and Social Inadaptation Assessment (MIA), *MDD* Major depressive disorder, *MEL* Munich Life Event List, *MINI* International Neuropsychiatric Interview, *NR* not reported, *OR* odds ratio, *PBI* Parental Bonding Instrument, *PRS*_*D*_ polygenic risk score for depression, *pT* *p*-value threshold, *Pseudo R²* goodness-of-fit measure for non-linear regression models, analogous to R² but not directly interpretable as explained variance, *r* correlation coefficient; *R²* proportion of variance in the outcome explained by the predictors, *RADS-2* Reynolds Adolescent Depression Scale, 2nd edition, *RERI* Relative Excess Risk due to Interaction, *SCL-90* Symptom-Checklist, *SDQ* Strengths and Difficulties Questionnaire, *SES* socioeconomic status, *SLE* Stressful life events, *sign.* significant, *SMFQ* The Short Mood and Feelings Questionnaire, *SRVS* Self-Report Victimization Scale, *TFMHQ* Thoughts and Feelings Mental Health Questionnaire, *WEMWBS* Warwick-Edinburgh Mental Well-Being Scale, *WEQ* War Events Questionnaire, *yrs* years, *YSR* Youth Self-Report questionnaires, *β* standardized regression coefficient, *≈* age not reported, inferred from the longitudinal design.

^1^a study was classified as “cross-sectional” when exposure and outcome were assessed at the same time point in the same sample, “retrospective longitudinal” when the exposure was measured before the outcome in time, but both were obtained from past records and analyzed after outcome occurrence, or as “prospective longitudinal” when both environmental exposures and depressive outcomes were prospectively assessed;

^2^data meta-analyzed in Peyrot et al. [27];

^3^final pT: *p*-value threshold which explained the highest variance in the respective study used in main analysis.

Moving beyond cross-sectional analyses, studies also investigated whether the interaction between PRS_D_ and CT prospectively predicts the onset of depressive symptoms. In one of the few studies on adolescents, Musci et al. [24] observed no interaction between PRS_D_ and early adversity (parental death and/or divorce by age 6) on state or trait internalizing symptoms in 488 participants, who were annually assessed over a seven-year period from late childhood ( ≈ 12 years) to late adolescence ( ≈ 18 years). Likewise, Halldorsdottir et al. [19] reported no PRS_D_ × CT interaction on MDD status, depressive symptoms and age of onset in both cross-sectional and longitudinal analyses across two independent cohorts. Instead, additive effects of PRS_D_ and CT (different types of abuse) on prospective depressive symptoms were found in an epidemiological cohort of 1450 adolescents that underwent follow-ups after 6, 12, and 24 months. In line with this finding, a cross-sectional analysis of data from a second adolescent sample comprising 279 MDD patients and 187 controls revealed comparable additive effects on MDD status and depression severity.

Complementing longitudinal GxE analyses in adolescent cohorts, three other studies investigated how PRS_D_ and retrospectively assessed CT interact to predict depressive symptoms in adulthood. In a sample of 516 soldiers who completed five assessments prior and up to 5 years after deployment, Schür et al. [28] reported no interaction between PRS_D_ and a broad spectrum of CT on baseline and post-deployment depressive symptoms. In contrast, significant additive and multiplicative interactions between PRS_D_ and CT (with an OR of up to 1.13) and between PRS_D_ and low parental bonding on depressive symptom trajectories over a fourteen-year period in adulthood were found in 3428 participants from the English Longitudinal Study of Ageing (ELSA, [20]). More precisely, GxE analysis revealed that the positive association between CT and depressive symptoms was stronger among those individuals with higher PRS_D_. In a subsequent wave of the ELSA study, the interaction between PRS_D_ and CT on depressive symptoms during the COVID-19 pandemic was not significant [30].

To conclude, while most studies suggest main effects of an individual’s PRS_D_ [19, 21], CT [23, 28–30] or both [20, 24, 26, 27, 31] on depression phenotypes, evidence for GxE interaction remains considerably heterogeneous. Notably, small but significant interactions between PRS_D_ and CT (including abuse, neglect, and early adversity) have been identified in the largest study to date [31] as well as in some smaller individual studies [20, 23, 26], with significant effects reported for both diagnosis of MDD [23, 26] and depressive symptoms [20, 31]. Overall, explained variance for significant GxE interactions was consistently very small (R² = 0.09–1.9%), standardized regression coefficients were modest (β ≈ 0.04), and observed odds ratios indicated only minor risk increases (OR = 1.13–1.16).

#### Other forms of childhood adversity and protective factors

A number of GxE studies have focused on other forms of childhood adversity associated with an increased risk for MDD. In a comprehensive study, Lehto et al. [35] analyzed the interaction of PRS_D_ and self-reported childhood adoption on depressive symptoms and other mental health outcomes in 243480 adult UKB participants. While the authors found no evidence for GxE interactions, each standard deviation increase in PRS_D_ was associated with an increase of 6% in the odds of being adopted, underlining the importance to account for potential genetic confounding in GxE studies. In contrast, a joint analysis of 2865 children and 38945 adults from the Lifelines Cohort Study revealed significant interactions between PRS_D_ and various stress-related exposures (including long-term difficulties, stressful life events, and lack of social support) on parent- and self-reported depressive symptoms [31], although explained variance was small (R² = 0.17%). When restricting the analyses to the child subsample, the GxE interaction effects vanished, while significant main effects of PRS_D_ and stress-related exposures remained.

Two longitudinal studies further investigated whether an individual’s PRS_D_ moderates the detrimental consequences of peer victimization. Among 748 adolescents from the Quebec Longitudinal Study of Children who were monitored twice over a two-year period, Perret et al. [37] reported no interaction between PRS_D_ and a multi-informant index of peer victimization on (prospective) depressive symptoms. Similar findings were obtained in the Avon Longitudinal Study of Parents and Children (ALSPAC) that provides data from 2268 participants assessed twice at age 13 and 23 years. In this study, an individual’s PRS_D_ and peer victimization were found to be independent predictors of depressive symptoms in early adulthood [33]. Interestingly, evidence for rGE was observed for self-reported, but not teacher-reported peer victimization [33, 37].

In contrast, findings from a six-year longitudinal study on 327 adolescents from the Research on Adolescent Development and Relationships (RADAR–Young) cohort suggested significant interactions between PRS_D_ and a multi-informant longitudinal index of critical parenting in predicting depressive symptom trajectories across adolescence [36]. Consistent with a diathesis–stress framework, latent growth curve modelling indicated that higher PRS_D_ was associated with elevated baseline depressive symptoms (β up to 0.15) and a stronger increase in symptoms over time (β up to 0.25), particularly among adolescents exposed to high parental criticism. Moreover, GxE effects varied depending on the *p*-value threshold applied for PRS_D_ construction, highlighting the sensitivity of results to whether the score included only genome-wide significant variants or also more liberal sets of variants with weaker associations to depression. Finally, this study also reported a significant gene–environment correlation, as higher PRS_D_ was positively associated with the likelihood of experiencing critical parenting.

Other studies focused on broader indicators of childhood adversity, namely (low) SES and educational attainment. In a register-based study comprising 17098 MDD patients and 18582 individuals randomly selected from the population, PRS_D_ and (low) childhood SES were again identified as independent predictors of early-onset depression [32]. Conflicting data stem from the ELSA study (n = 6202), which followed older adults over 14-year period [34]. Here, a weak but significant interaction between PRS_D_ and educational attainment was observed for baseline depressive symptoms in late life (β = –0.01), indicating that each additional year of completed schooling predicted a slight reduction in depressive symptoms among those with higher genetic liability for depression. No interaction effects were found for changes in depressive symptoms over time.

Further research into protective factors in addition to educational attainment, was performed in one cross-sectional analysis of 35633 participants from the UKB [17]. As reported above, this study found no significant GxE effect for CT. However, a significant interaction emerged between PRS_D_ and the experience of feeling loved as a child on age of depression onset (β = 0.708), which was particularly pronounced in males (β = 1.421). These findings highlight the importance of incorporating positive as well as negative environmental factors in GxE research on depression. A subsequent GWEIS identified a novel candidate locus interacting with felt loved as a child (*GSAP*) as well as several genes with suggestive significant associations (*CMYA5*, *KIRREL3*) in males. Another study exploring protective factors in 4975 children participating in the ABCD study, however, reported independent effects of (lack of) sport activity and PRS_D_ on anxious/depressed, withdrawn/depressed symptoms, with no evidence for GxE [82].

Consistent with findings on CT, a higher PRS_D_ and different forms of childhood adversity were identified as independent predictors of depressive phenotypes in most studies [32, 33, 35, 37], with substantial evidence for gene–environment correlation [32, 33, 35–37]. Significant, albeit weak GxE effects have been reported with critical parenting (β up to 0.25) and low educational attainment (β = –0.01) [34, 36]. With respect to protective factors, only few studies exist [17, 31] but first evidence suggests significant interactions, such as with social support (β = –0.0417; R² = 0.09%) and the experience of feeling loved as a child (β = 0.708; in males up to β = 1.421).

#### Prenatal adversity

While most studies investigated adversity in early childhood, others have begun to examine prenatal exposures given the extensive literature on developmental origins of psychiatric symptoms [83]. In a prospective longitudinal analysis within ALSPAC (n = 5546), Chen et al. [43] tested whether a child’s PRS_D_ moderates effects of prenatal maternal depression/anxiety on internalizing symptoms from ages 4–16. While maternal depression and anxiety as well as a child’s PRS_D_ independently and additively predicted internalizing symptoms, the authors found no evidence for a significant GxE interaction. However, weak correlations between a child’s PRS_D_ and maternal psychopathology were observed, indicating significant rGE. Respective findings were replicated in the Prevention of Preeclampsia and Intrauterine Growth Restriction cohort (n = 514 children) that provided complementary measures. Another study focused on maternal vitamin D status during gestation, defined by circulating levels of 25-hydroxyvitamin D [25(OH)D], that has been previously linked to the child’s neurodevelopment and mental health status [84]. Analyzing data from 2938 mother-child dyads from the ALSPAC, Wang et al. [42] found no evidence for an interaction of PRS_D_ and prenatal vitamin D status on depression risk in childhood and adolescence.

So far, only few studies have examined whether PRS_D_ interacts with heterogeneous prenatal exposures. Available evidence points to independent main effects of PRS_D_, maternal depressive and anxiety symptoms, and (to a lesser extent) maternal vitamin D status on child mental health, but no robust GxE interactions [42, 43].

### Studies investigating the interaction between PRS_D_ and adult or cumulative environmental exposures on depression-related outcomes

#### Trauma and stressful life events in adulthood

Most studies on environmental exposures in adulthood (Table 2) focused on the interaction of PRS_D_ and different types of trauma or SLEs implicated in the pathogenesis of MDD [85]. In a first cross-sectional study on 8761 older adults from the Health and Retirement Study (HRS), Musliner et al. [49] reported additive main effects of an individual’s PRS_D_ and SLEs in the past two years on depressive symptoms, but no GxE interaction. Comparable findings were obtained from analyzing cross-sectional data of 1605 MDD patients and 1064 controls from the RADIANT UK cohort [23]. In order to account for a significant rGE of PRS_D_ and adult SLEs observed in this study, the authors conducted separate GxE analyses for those SLEs dependent on (e.g., unemployment) or independent of (e.g., death or illness of a family member) the individual’s behavior, both yielding no additive or multiplicative interaction with PRS_D_ on MDD status. By contrast, in another cross-sectional study comprising 4919 individuals from the Generation Scotland Cohort, a higher PRS_D_ was found to increase depressive symptoms in those participants reporting high numbers of SLE over the past 6 months [44]. The respective GxE interaction was nominally significant in the full cohort, explaining 0.08% of the variance in depression. Stratified analyses revealed significance in women, but not in men. Convergent evidence for significant interactions of PRS_D_ and a broad range of stress-related exposures on interview-derived depressive symptoms stems from the Lifelines Cohort Study (n = 41810). Here, the effect of an individual’s PRS_D_ was significantly amplified by self-reported SLEs, long-term difficulties and lack of social support, with GxE effects explaining up to 0.17% of the variance [31]. GxE effects attenuated after adjusting for the interactions between SES and stress-related exposures in order to account for small rGEs observed in this study. When restricted to adults, GxE effects remained significant and explained up to 0.15% of the variance in depressive symptoms. Extending this line of research, Colodro-Conde et al. analyzed cross-sectional data from 5221 twins [54] and explicitly differentiated between personal SLEs and those experienced by an individual’s close network in the past 12 months. Results suggested a multiplicative interaction of PRS_D_ and personal SLEs (but not network SLEs) that contributed positively to the risk of depression, yet explaining only a small amount of variance (0.12%). Respective GxE interaction was predominantly driven by women and by those personal SLEs that depend on the individual’s behavior (that were also found to be more heritable), raising concerns that obtained findings could be partly attributed to rGE.

**Table 2:** Studies investigating the interaction between a PRS_D_ and adult or cumulative environmental adversity on depression-related outcomes.

author	study design^1^	population (ancestry)	age (mean)	female (%)	PRS discovery sample	PRS p-value threshold (pT)	environmental exposure	predicted outcome	main results (effect size estimate [CI])
*Trauma and stressful life events*									
Arnau-Soler et al., [11]	cross-sectional	4919 (European)	57.2 yrs	60.8	PGC-MDD-2013, July 2016* (excluding 23andMe & Generation Scotland data)	8 pTs tested (range: <5x10^-8^ to <=1)	SLEs in the past 6 months (TSLE)	depressive symptoms (GHQ)	• nominally sign. interaction of PRS_D_ and SLEs (best-fit pT: 0.01) on depressive symptoms (largest β = 0.028, R² = 0.08%), sign. in women (β = 0.044, R² = 0.19%), not men (β = 0.039, R² = 0.15%) • sign. GxE interactions for both active (largest β = 0.039, R² = 0.15%) and passive SLEs (largest β = 0.040, R² = 0.16 %) in women • sign. main effects of PRS_D_ (β = 0.080, largest R² = 0.64%) and SLEs (β = 0.222, R² = 4.91%) • sign. correlation of PRS_D_ and SLEs (largest R² = 0.40-0.45%^v^)
Choi, & Chen et al., [45]	prospective longitudinal	3079 soldiers (European)	pre-deployment t1 = 25.9 yrs post-deployment t2 ≈ 26.3 yrs t3 ≈ 27.2 yrs	4.0	PGC-MDD-2018 (excluding 23andMe data)	8 pTs tested (range: 5x10^-8^ to 1.0; final pT: 0.01)	combat stress exposure (self-report); unit cohesion (WRAIR MCS)	MDD post- deployment (CIDI-SC based self-report)	• no sign. interaction of PRS_D_ and unit cohesion or combat stress on incident MDD (largest OR = 1.32 [0.96,1.76]) • sign. main effects of PRS_D_ (largest OR = 1.58 [1.12, 2.25], Pseudo R^2^ = 0.4%), unit cohesion (largest OR = 0.67 [0.60, 0.74]) and combat stress (largest OR = 1.34 [1.21, 1.50]) • no sign. correlation of PRS_D_ and unit cohesion (r = −0.02)
Colodro-Conde et al., [54]	cross-sectional	5221 twins (European)	35.7 yrs	65.6	PGC-MDD-2013, July 2016* (excluding 23andMe & QIMR data)	8 tested (range: <5x10^-8^ to p<1; final pT: p<1)^2^	personal and network SLEs in the past 12 months (HLQ)	depressive symptoms (DSSI & SCL-90 based)	• sign. interaction of PRS_D_ and personal SLEs on depressive symptoms in women (largest R² = 0.12%), not men • sign. main effects of PRS_D_ (largest R² = 0.46%), personal SLEs (R² = 12.9%) and network SLEs (R² = 0.3%) • sign. correlation of PRS_D_ and SLEs (NR)
Domingue et al., [46]	prospective longitudinal	8588 (non-Hispanic white)	t1 = 59 yrs t2 ≈ 60 yrs	58.0	PGC-MDD-2013 & SSGAC-2016	NR	death of spouse (self-reported)	depressive symptoms (CES-D)	• sign. interaction of PRS_D_ (SSGAC & PGC based) and death of spouse on depressive symptoms (largest β = 0.69 [0.24, 1.14]) • sign. main effects of PRS_D_ (SSGAC & PGC based, largest β = 0.13) and death of spouse (β = 1.97 [1.50, 2.40])
Fang et al., [47]	prospective longitudinal	5227 medical interns (European)	pre-internship t1 = 27.6 yrs during internship t2 ≈ 27.9 yrs t3 ≈ 28.1 yrs t4 ≈ 28.4 yrs t5 ≈ 28.7 yrs	50.3	MDD-2019 (excluding 23andMe data)	9 pTs tested (range: <5 ×10^−8^ to <0.5)	medical internship stress	MDD, depressive symptoms (PHQ-9)	• sign. interaction of PRS_D_ and internship stress on depressive symptoms (largest β = 0.036) and MDD diagnosis (OR = 1.40; RERI = 31.75 [5.47, 58.03]) • sign. main effects of PRS_D_ (β = 0.095; OR = 1.21) and internship stress (NR)
Mullins et al., [23]	cross-sectional	1605 MDD 1064 HC (European)	36.7 yrs 41.5 yrs	70.7 59.0	PGC-MDD-2013 (excluding RADIANT UK data)	9 pTs tested (range: <0.0001 to <0.5)	adult SLEs (LEQ-B)	depression (SCAN)	• no sign. additive (largest OR = 1.01 [0.99, 1.02], R² = 0.07%) or multiplicative (largest OR = 1.05 [0.98, 1.12], R² = 0.1%) interaction of PRS_D_ and SLEs (for both dependent and independent SLEs) on MDD status • sign. main effects of PRS_D_ (OR = 1.22 [1.12, 1.32], R² = 1.1%) and SLEs (dependent: R² = 6.6%, independent: R² = 1.9%, total: R² = 0.7%) • sign. correlation of PRS_D_ and dependent SLEs in MDD patients (OR = 1.08 [1.03, 1.14])
Musliner et al., [49]	cross-sectional	8761 (mostly European)	63.9 yrs	62.0	PGC-MDD-2013	10 pTs tested (range: <0.10 to <1.0; final pT: <0.4)^2^	SLEs in the past 2 yrs (self-report)	depressive symptoms (CES-D)	• no sign. interaction of PRS_D_ and SLEs on depressive symptoms (CES-D score ≥ 4, β = −0.06) and number of symptoms (β = −0.0003, Pseudo R² = 2.01%) • sign. main effects of PRS_D_ (β = 0.21, Pseudo R² = 0.95%) and SLEs (OR = 2.19 [1.86, 2.58], Pseudo R^2^ = 1.08%) • no sign. correlation of PRS_D_ and SLEs (β = 0.02)
Peter et al., [52]	prospective longitudinal	218 stressed students (European)	22.9 yrs (6 assessments within 13 months)	73.0	PGC-MDD & 23andMe 2019	≤0.05	exam period	depressive symptoms (HADS), perceived stress (self-report)	• no sign. interaction of PRS_D_ and exam stress on depressive symptoms (b = −0.05) or perceived stress (b = 0.04) • no sign. 3-way interactions with time on depressive symptoms (b = 0.20) or perceived stress (b = 0.02) • no main effects of PRS_D_ (largest b = 0.07) and exam stress (largest b = 0.09), but sign. interaction of exam stress x time on depressive symptoms (b = 0.50) and perceived stress (b = 0.18)
		214 controls (European)	21.0 yrs (6 assessments within 13 months)	77.0					
Schür et al., [28]	prospective longitudinal	516 soldiers (European)	t1 = 29.6 yrs t2 ≈ 29.7 yrs t3 ≈ 30.2 yrs t4 ≈ 31.2 yrs t5 ≈ 32.2 yrs	8.5	PGC-MDD-2018	13 pTs tested (range: <5x10^-8^ to <1)	deployment-related trauma (self-report)	post-deployment depressive symptoms (SCL-90)	• no sign. interaction of PRS_D_ and deployment-related trauma on post-deployment depressive symptoms (largest β = −0.3087) • no sign. main effects of PRS_D_ (largest β = −4.4056) and deployment-related trauma (NR) • nominally sign. correlation of PRS_D_ and deployment-related trauma (b = −0.49)
Turner et al., [50]	prospective longitudinal	331 college freshmen (European)	18-19 yrs (up to 13 assessments within 1 yr)	58.4	PGC-MDD & 23andMe 2019	=1.0	Covid-19 pandemic stress	depressive symptoms (PHQ-9)	• sign. interaction of PRS_D_ and Covid-19 pandemic stress on depressive symptoms throughout the first freshman year (NR) • sign. main effects of PRS_D_ (β = 0.521) and Covid-19 pandemic stress (NR)
Wang et al., [31]	cross-sectional	overall sample: 41810 (European)	42.0 yrs	58.9	PGC-MDD & 23andMe 2019	11 pTs tested (range: <5 x 10^-8^ to <1)	long-term difficulties (LDI); SLE (LTE); social support (SPF-IL)	depressive symptoms (MINI)	overall sample: • sign. interaction of PRS_D_ and long-term difficulties (largest β = 0.0541, R² = 0.17%), SLE (largest β = 0.0385, R² = 0.05%) and social support (largest β = −0.0417, R² = 0.09%) on depressive symptoms • sign. main effects of PRS_D_ (β = 0.1105, R² = 0.66%), long-term difficulties, SLE and social support (largest β = 0.4195, R² = 9.54%) • sign. correlation of PRS_D_ and long-term difficulties, SLEs, social support (largest r = 0.08)
		adult subsample: 38945 (European)	44.1 yrs	59.6					adult subsample: • sign. interaction of PRS_D_ and long-term difficulties (largest β = 0.0515, R² = 0.15%), SLE (largest β = 0.0324, R² = 0.06%) and social support (largest β = −0.0423, R² = 0.07%) on depressive symptoms • sign. main effects of PRS_D_ (β = 0.1149, R² = 0.69%), long-term difficulties, SLE and social support (largest β = 0.4350, R² = 10.19)
*Adverse and protective social environments in adulthood*									
Cleary et al., [51]	prospective longitudinal	1011 medical interns (European)	pre-internship t1 = 27.6 yrs during internship t2 ≈ 27.9 yrs t3 ≈ 28.1 yrs t4 ≈ 28.4 yrs t5 ≈ 28.7 yrs	47.6	PGC-MDD & 23andMe 2019 (MDD-PGC2 + 23andMe)	NR	changes in social support during internship (MSPSS)	depressive symptoms (PHQ-9)	• sign. interaction of PRS_D_ and changes in social support on depressive symptoms during internship (IRR = 0.96 [0.93, 0.98]) • sign. main effects of PRS_D_ (IRR = 1.14 [1.04,1.24]) and changes in social support (IRR = 0.88 [0.86–0.90])
		435 widows (European)	t1 = 75.6 yrs (follow-up after loss of a spouse)	70.8			changes in social support after loss of a spouse (LBQ)	depressive symptoms (CES-D)	• sign. interaction of PRS_D_ and changes in social support on depressive symptoms after loss of a spouse (IRR = 0.78 [0.66, 0.92]) • no sign. main effects of PRS_D_ (IRR = 1.08 [0.96, 1.22]) and changes in social support (IRR = 1.04 [0.89, 1.22])
Colodro-Conde et al., [54]	cross-sectional	5221 twins (European)	35.7 yrs	65.6	PGC-MDD-2013, July 2016* (excluding 23andMe & QIMR data)	8 tested (range: <5 x 10^-8^ to p < 1; final pT: p < 1)^2^	perceived social support (KPSS)	depressive symptoms (DSSI & SCL-90 based)	• sign. interaction of PRS_D_ and (lack) of social support (largest R² = 0.07%^v^) • sign. main effects of PRS_D_ (largest R² = 0.46%) and (lack) of social support (R² = 3%) • sign. correlation of PRS_D_ and SLEs (NR)
Giannelis et al., [55]	cross-sectional	52078 (European)	63.6 yrs	52.0	PGC-MDD-2018 (excluding 23andMe and UK Biobank data)	11 pTs tested (range: <5 x 10^-8^ to <1; final pT: 0.3)^2^	family status: living with spouse/partner, number of children (self-report)	lifetime depression (CIDI based self-report)	• no sign. interaction of PRS_D_ and family status on lifetime depression (NR) • sign. main effects PRS_D_ (NR) and family status (largest OR = 1.27 [1.14, 1.42]) • no sign. correlation between PRS_D_ and family status (largest OR = 1.006 [0.99, 1.01])
Steen et al., [56]	prospective longitudinal	19128 (mostly European)	52.4 yrs (16 assessments within 1 yr)	62.3	MDD-2019	<0.05	loneliness (UCLA-L)	MDD (DSM-criteria based diagnostic interviews), depressive symptoms (MINI)	• no sign. interaction between PRS_D_ and loneliness on MDD (OR = 0.978 [0.950, 1.006]) and depressive symptoms (IRR = 0.994 [0.985, 1.002]) • sign. main effect of PRS_D_ on MDD (OR = 1.265 [1.151, 1.391]) and on depressive symptoms (IRR = 1.116 [1.087, 1.145]) • sign. main effect of loneliness on MDD (OR = 1.539 [1.482, 1.598]) and depressive symptoms (IRR = 1.271 [1.225, 1.320])
Stringa et al., [57]	prospective longitudinal	cohort 1 (European): 590	t1 = 70.5 yrs (+23 yrs follow-up every 3 yrs)	50.7	PGC-MDD-2018 (excluding 23andMe data)	<0.05 (main analysis) & <0.2 (sensitivity check)	partner status, network size, emotional support (self-report)	depressive symptoms (CES-D)	Meta-analysis across cohorts: • no sign. interaction of PRS_D_ and partner status (b = −0.014 [−0.077, 0.049]), network size (b = −0.001 [−0.003, 0.002]), and emotional support (b = 0.001 [−0.002, 0.003]) on depressive symptoms over time • sign. main effects of PRS_D_ (b = 0.053 [0.023, 0.083]), partner status (b = −0.325 [−0.483, −0.166]), network size (b = −0.008 [−0.010, −0.005]), no sign. main effect of social support (b = 0.001 [−0.002, 0.003]) • no sign. correlation of PRS_D_ and a) partner status, b) network size, c) emotional support (NR)
		cohort 2 (European): 491	t1 = 68.8 yrs (+23 yrs follow-up every 3 yrs)	56.2					
		cohort 3 (European): 631	t1 = 60.0 yrs (+13 yrs follow-up every 3 yrs)	52.8					
		cohort 4 (European): 567	t1 = 60.5 (+3 yrs follow-up)	51.5					
*Other adverse and protective environmental exposures in adulthood*									
Cao et al., [58]	prospective longitudinal	339767 (European)	t1: 56.6 yrs (median follow-up of 8.1 yrs)	52.5	PGC-MDD-2013	none (PRS-CS)	healthy lifestyle factors (self-report)	depression (clinical records)	• no sign. interaction of PRS_D_ and healthy lifestyle (t1) factors on the incident of depression (largest HR = 1.03 [0.81, 1.32]) • sign. main effects of PRS_D_ (HR = 1.22 [1.14, 1.30]) and healthy lifestyle factors (largest HR = 3.52 [3.22, 3.84])
Chen & Yang et al., [60]	retrospective longitudinal	247828 (European)	t1: 53.0 yrs (median follow-up of 12.7 yrs)	50.9	GERA-2018	none (PRS-CS)	night shift work (self-report)	MDD (clinical records, self-report)	• no sign. interaction of PRS_D_ and night shift work on risk of MDD during follow-up (additive: RERI: −0.017 [−0.326, 0.292], multiplicative: NR) • sign. main effects of PRS_D_ (HR = 1.14 [1.081.21]) and night shift work (largest HR = 1.32 [1.20, 1.45])
Choi, & Zheutlin et al., [59]	prospective longitudinal	7968 Health Care patients (European)	t1 ≈ 57.9 yrs t2 = 59.9 yrs	57.0	MDD-2019 (excluding 23andMe data)	10 pTs tested (range: 5 x 10^-8^ to 1.0; final pT: 1.0)^2^	physical activity in the past year (self-report)	MDD (ICD-9/10 code retrieved from patient data registry)	• no sign. interaction of PRS_D_ and physical activity (t1) on incident MDD within a two-year time period (OR = 1.05 [0.96, 1.15]) • sign. main effects of PRS_D_ (OR = 1.20 [1.11, 1.31]) and (lack of) physical activity (OR = 0.83 [0.75, 0.90]) • no sign. correlation of PRS_D_ and physical activity (r = 0.006)
Fu et al., [62]	prospective longitudinal	14189 MDD 476591 controls (European)	56.4 yrs 56.6 yrs (median follow-up of 8.8 yrs)	63.8 54.1	PGC-MDD & 23andMe 2019	NR (97 SNPs included)	air pollution (geographic information systems)	MDD (clinical records)	• sign. multiplicative (NR) and additive interaction of PRS_D_ and air pollution on MDD (largest RERI = 0.15 [0.07, 0.23]) • sign. main effects PRS_D_ (HR = 2.75 [2.43, 3.12]) and air pollution (largest HR = 2.12 [1.82, 2.47])
Kosciuszko et al., [34]	retrospective longitudinal	6202 (British)	65.2 yrs (5 follow-ups over 14 yrs)	52.2	PIR-2021	NR	SES (wealth in adulthood)	depressive symptoms (CES-D)	• sign. interaction of PRS_D_ and (low) wealth on baseline depressive symptoms (largest β = 0.08 [0.03, 0.13]), but not on rate of change in depressive symptoms (largest β = −0.002 [−0.01, 0.00]) • sign. main effects of PRS_D_ (β = 0.12 [0.08, 0.17]) and (low) wealth on baseline depressive symptoms (β = 0.77 [0.71, 0.83]) • no sign. main effects of PRS_D_ (β = −0.004 [−0.02, 0.12]) and low wealth (β = −0.02 [−0.05, 0.001]) on rate of change in depressive symptoms
Lin et al., [61]	retrospective longitudinal	13636 MDD 367340 controls (European)	57.1 yrs 56.8 yrs (continuously followed for up to ≈ 12.6 yrs)	61.7 52.7	GERA-2018	none (PRS-CS)	time spent in outdoor light (self-report)	MDD (clinical records)	• sign. interaction of PRS_D_ and time spent in outdoor light on MDD (largest HR = 1.05 [0.90, 1.22]) • sign. main effects of PRS_D_ (HR: 1.08 [1.06, 1.09]) and time spent in outdoor light (largest HR = 1.13 [1.07–1.20])
Sund et al., [88]	cross-sectional	41198 (Norwegian)	54.4 yrs	56.2	MDD-2019	<5 x 10^-8^	urbanicity (secondary ecological data)	depressive and anxious symptoms (HADS), mental distress (MHI)	• no sign. interaction of PRS_D_ and urbanicity on depressive symptoms (largest OR = 0.91 [0.79, 1.05]) and other mental health outcomes • no sign. main effects of PRS_D_ (largest OR = 1.09 [1.00, 1.18]), sign. main effect of urbanicity (largest OR = 1.34 [1.15, 1.56]) • sign. correlation of PRS_D_ and urbanicity (NR)
Wesseldijk et al., [63]	cross-sectional	5648 (European)	40.4 yrs	59.1	PGC-MDD & 23andMe 2019	NR	music engagement & achievement (CAQ); lifetime amount of music practice (self-reported)	depression (registry-based ICD-10 diagnosis), depressive symptoms (HSC)	• no sign. interaction of PRS_D_ and music engagement or music practice on depression/depressive symptoms (NR) • sign. main effects of PRS_D_ (HR = 1.43) on depression and depressive symptoms (β = 0.08) and of music engagement on depression (HR = 1.25) and depressive symptoms (β = 0.08) • no sign. main effects of music practice or achievement on depression (largest HR = 1.09), but sign. main effect of music practice on depressive symptoms (β = 0.04) • sign. correlation of PRS_D_ and music engagement (OR = 1.08) and music practice (β = 0.04)
*Cumulative lifetime adversity*									
Coleman et al., [64]	cross-sectional	35269 MDD 63451 HC (European)	64.1 yrs	56.3	PGC-MDD-2018 (excluding UK Biobank data, including 23andMe)	7 pTs tested (range: <0.001 to <0.5; final pT: 0.5)^2^	lifetime trauma exposure (self-report)	MDD (CIDI based self-report)	• sign. additive (β = 0.011 [0.008, 0.014]), but not multiplicative (OR = 1.01 [1.00, 1.03]) interaction of PRS_D_ and lifetime trauma on MDD risk • sign. main effect of PRS_D_ (largest OR = 1.26 [1.24, 1.28]), main effect of lifetime trauma NR • sign. correlation of PRS_D_ and lifetime trauma (NR)
Lipsky et al., [70]	cross-sectional	1389 veterans	36.4 yrs	17.4	PGC-MDD-2018	1001 PTs tested (range p= 0.0001 to 1); final pT: NR	lifetime trauma (TLEQ)	MDD (SCID-I)	• no sign. interaction of PRS_D_ and lifetime trauma on MDD (largest OR = 1.03 [0.90, 1.19]) • sign. main effects of PRS_D_ (largest OR = 1.19 [1.01, 1.39]) and lifetime trauma (OR = 1.22 [1.06, 1.41])
Musliner et al., [68]	retrospective longitudinal	cohort 1 (European) 18532 MDD	10 yrs (continuously followed for up to 21 yrs)	68.5	MDD-2019	=1.0	SLEs (population-based registers)	early-onset depression (clinical records)	overall sample: • sign. multiplicative interaction of PRS_D_ and SLEs on early-onset depression (HR = 0.96 [0.94, 0.99]) • sign. additive interaction of PRS_D_ and SLEs on early-onset depression (opposite effect, RERI = 0.09 [0.06, 0.12]) • sign. main effects of PRS_D_ (HR = 1.35 [1.31, 1.38]) and SLEs (HR = 1.36 [1.33, 1.39]) • sign. correlation of PRS_D_ and SLEs (HR = 1.09 [1.07, 1.11])
		cohort 2 (European) 20184 controls		49.2					
Peterson et al., [66]	cross-sectional	4785 MDD 4814 controls (Han Chinese)	45.3 yrs 47.7 yrs	100.0	PGC-MDD-2013	<0.2	lifetime trauma/SLE (self-report)	MDD (CIDI based self-report)	• no sign. additive (b = 0.002 [0.000, 0.005], Pseudo R² = 0.03%) and multiplicative (OR = 1.009 [1.000, 1.019], Pseudo R² = 0.06%) interaction between PRS_D_ and lifetime trauma/SLE on MDD risk • no sign. main effect of PRS_D_ (OR = 1.004 [1.000, 1.009], largest Pseudo R² = 0.15%), sign. main effect of lifetime trauma/SLE (OR = 6.811 [2.091, 22.293], largest Pseudo R² = 3.13%) • no sign. correlation between PRS_D_ and lifetime trauma/SLE (largest OR = 1.005 [0.999, 1.011], Pseudo R² = 0.07%)
Thorp et al., [69]	cross-sectional	102182 (European)	56.1 yrs	56.0	PGC-MDD-2018	NR	lifetime trauma (self-report)	current MDD, depressive symptoms (PHQ-9); lifetime MDD (CIDI based self-report)	• sign. additive interaction of PRS_D_ and lifetime trauma on current MDD (b = 0.158 [0.111, 0.205]), lifetime MDD risk (b = 0.015 [0.008, 0.022]), and six out of nine current depressive symptoms (largest b = 0.026 [0.018, 0.034]) • no sign. multiplicative interaction of PRS_D_ and lifetime trauma on current MDD (OR = 0.996 [0.964, 1.029]), lifetime MDD risk (OR = 1.009 [0.973, 1.047]), and six out of nine current depressive symptoms (largest OR = 1.010 [0.983, 1.036]) • no sign. interaction of PRS_D_ and lifetime trauma on specific lifetime MDD symptoms (additive: b = 0.009 [−0.003, 0.022]; multiplicative: largest OR = 1.113 [0.973, 1.273]) • sign. main effects of PRS_D_ (largest OR = 1.247 [1.226, 1.269]) and lifetime trauma (OR = 4.627 [4.465, 4.795])

*b* unstandardized regression coefficient, *CAQ* Creative Achievement Questionnaire, *CES-D* Center for Epidemiologic Studies Depression Scale, *CI* confidence interval, *CIDI* Composite International Diagnostic Interview, *CIDI-SC* Composite International Diagnostic Interview Screening Scale, *DSM-criteria* Diagnostic and Statistical Manual of Mental Disorders, *DSSI* Delusions-Symptoms-States Inventory, *GHQ* General Health Questionnaire, *HADS* Hospital Anxiety and Depression Scale, *HC* healthy controls, *HLQ* Health and Lifestyle questionnaire, *HR* hazard ratio, *HSC* Hopkins Symptom Checklist, *ICD-9/10* International Classification of Diseases, 9th and 10th revision, *IRR* incidence rate ratio, *KPSS* Kessler Perceived Social Support Measure, *LBQ* Leave-Behind Questionnaire, *LDI* Long-term Difficulties Inventory, *LEQ-B* Brief Life Event Questionnaire, *LTE* List of Threatening Events, *MDD* major depressive disorder, *MHI* Mental Health Index, *MINI* Mini-International Neuropsychiatric Interview, *MSPSS* Multidimensional Scale of Perceived Social Support, *NR* not reported, *OR* odds ratio, *PHQ-9* depression subscale of the Patient Health Questionnaire (PHQ), *PRS*_*D*_ polygenic risk score for depression, *PRS-CS* polygenic prediction method using continuous shrinkage (CS) priors, *pT* *p*-value threshold, *Pseudo R²* goodness-of-fit measure for non-linear regression models, analogous to R² but not directly interpretable as explained variance, *r*  correlation coefficient, *R²* proportion of variance in the outcome explained by the predictors, *RERI* Relative Excess Risk due to Interaction, *SCAN* Schedules for Clinical Assessment in Neuropsychiatry Interview, *SCID-I* Structured Clinical Interview for DSM-IV-TR Axis I Disorders, *SCL-90* Symptom Checklist-90, *SES* socioeconomic status, *sign*. significant, *SLE* stressful life events, *SPF-IL* Social Production Function Instrument for the Level of Well-being, *t* timepoint, *TLEQ* Traumatic Life Events Questionnaire, *TSLE* total number of stressful life events, *UCLA-L* short version of Revised UCLA (University of California, Los Angeles) Loneliness Scale, *WRAIR MCS* Walter Reed Army Institute of Research (WRAIR) Military Cohesion Scales, *yr* year, *yrs* years, *β* standardized regression coefficient.

^1^a study was classified as “cross-sectional” when exposure and outcome were assessed at the same time point in the same sample, “retrospective longitudinal” when the exposure was measured before the outcome in time, but both were obtained from past records and analyzed after outcome occurrence, or as “prospective longitudinal” when both environmental exposures and depressive outcomes were prospectively assessed;

^2^final pT: *p*-value threshold which explained the highest variance and is therefore used in the main analysis;

^v^visual inspection.

A number of studies further applied a pre-post design to investigate GxE interactions on prospective depressive symptom development after exposure to traumatic/stressful events. A first study on 516 soldiers reported no evidence for an interaction of PRS_D_ and deployment-related trauma on post-deployment depressive symptoms [28]. Comparable results stem from 3079 soldiers participating in the Army Study of Risk and Resilience in Servicemembers (STARRS) that focused on combat stress but also on protective effects of unit cohesion, where no interactions with PRS_D_ on the incidence of MDD were observed [45]. Besides war stress, other prospective longitudinal studies focused on students during periods of chronic stress exposure in an academic setting. In a sample of 432 law students preparing for their first state examination and controls, an individual’s PRS_D_ was found to be unrelated to trajectories of depressive symptoms and perceived stress over a 13-month period [52]. In another study on 311 college freshmen, however, Turner et al. reported associations between PRS_D_ and depressive symptoms throughout the first freshman year during the COVID-19 pandemic as compared to a pre-pandemic cohort [50]. While lower PRS_D_ predicted a reduced risk for depression during a typical freshman year, no associations were observed during the Covid-19 pandemic, indicating that this genetic advantage may vanish under specific, e.g., unfamiliar stress conditions. Partly conflicting results were obtained in the Intern Health Study comprising 5227 medical interns, where an individual’s PRS_D_ was more strongly associated with depressive symptoms (β up to 0.036) and MDD diagnosis (OR = 1.40) in the aftermath of physician training stress than at baseline [47]. Likewise, in another longitudinal analysis from the HRS cohort on 8588 older adults, Domingue et al. [46] found that individuals with lower PRS_D_ were less likely to develop depressive symptoms following the death of their spouse (β up to 0.69).

In summary, we observed substantial evidence for interactions of PRS_D_ and adult trauma/SLEs on both depressive symptoms [31, 44, 46, 47, 50, 54] and, to a lesser extent, depression diagnosis [47], while other studies suggest their main effects to be additive [23, 45, 49]. Across studies, significant GxE effects were consistently small in magnitude, with standardized regression coefficients ranging from β = 0.028 to 0.69, explained variance from R² = 0.05–0.15%, and a single OR of 1.40.

#### Adverse and protective social environments in adulthood

We further retrieved studies that investigated the interplay of PRS_D_ and an individual’s social environment, including negative (e.g., network stressors) and positive (e.g., social support) aspects. Colodro-Conde et al. [54], whose cross-sectional study of 5221 twins has been described above, examined not only personal and network SLEs but also perceived social support, and observed a multiplicative interaction of PRS_D_ with social support (R² ≈ 0.07%). Merging data from the HRS (n = 435) and the Intern Health Study (n = 1011), Cleary et al. further investigated whether depressive symptoms following developmentally normative life stressors are moderated by self-reported changes in social support [51]. In both cohorts, an individual’s PRS_D_ was found to interact with changes in social support on depressive symptoms following death of a spouse (IRR = 0.78) or physician training stress (IRR = 0.96). Precisely, individuals with highest PRS_D_ were most sensitive to a loss of social support in terms of depressive symptoms, but also benefited the most from gaining social support. These findings align with a differential susceptibility framework, where factors that confer risk in negative environments also confer benefits in positive environments [86]. Two other longitudinal studies also showed no evidence of GxE interactions with protective social factors. Stringa et al. [57] meta-analyzed prospective data from four cohorts of the Longitudinal Aging Study of Amsterdam (n = 2279) that included repeated assessments of depressive symptoms over 3–23 year periods and reported independent, but no interaction effects of PRS_D_ and partner status, social network size or emotional support. Likewise, analyzing data from 52078 UKB participants revealed no moderating effect of an individual’s family status (living with spouse/partner, number of children) on the association of PRS_D_ and lifetime depression [55]. Finally, in the Lifelines COVID-19 study, Steen et al. [56] performed a high-resolution assessment of subjective feelings of loneliness, a well-known risk factor for mental health problems [53], and depressive symptoms within one year during the pandemic. While both PRS_D_ and loneliness significantly predicted depressive symptoms and MDD diagnosis during the pandemic, again, no GxE interaction occurred.

To conclude, two studies reported significant interactions of PRS_D_ with aspects of the social environment (perceived social support: [54]; changes in social support: [51]), yet the majority of studies found additive rather than interactive effects [55–57]. Where significant, interaction effects were consistently small in magnitude (R² = 0.07%; IRRs ≈ 0.78–0.96).

#### Other adverse and protective environmental exposures in adulthood

A number of GxE studies have focused on other forms of environmental exposures implicated in the pathogenesis of MDD, including both protective (e.g., healthy lifestyle) and risk (e.g., urbanicity) factors.

A first longitudinal study utilizing electronic health records from 7968 older adults revealed no interaction between physical activity in the past year, a likely causal factor in reducing MDD risk, and an individual’s PRS_D_ on the diagnosis of MDD within a two-year period [59]. Comparable results were obtained in a prospective analysis of 339767 UKB participants who provided information on multiple healthy lifestyle factors, such as regular physical activity, healthy diet and moderate alcohol intake [58]. In this study, a combined healthy lifestyle score and an individual’s PRS_D_ were independently associated with incident depression during a follow-up of 8 years, with no evidence for GxE interaction. Likewise, an individual’s PRS_D_ and working night shifts were found to be independent predictors of a higher risk of depression in 247828 UKB participants after a median follow-up of 12.7 years [60]. In contrast, an average of 1.5 h/day spent in outdoor light predicted lower MDD risk in 380976 UKB participants during a 12.6 year time interval independent of PRS_D_ [61]. Here, evidence for GxE interaction was restricted to intermediate PRS_D_ levels (HR up to 1.05), indicating a small effect size. Another study on 5648 Swedish twins further reported significant main effects of music engagement and PRS_D_ on depression/depressive symptoms, with no evidence for GxE interaction [63].

Based on an ongoing discussion regarding urban-rural differences in mental health conditions [87], two other cross-sectional studies analyzed PRS_D_ interactions with urbanicity and air pollution. Utilizing secondary ecological data from 41198 participants of the Nord-Trøndelag Health study (HUNT), Sund et al. [88] observed that rural residents had increased odds for reporting depressive symptoms, independent of an individual’s PRS_D_. Consistent with previous reports of rGE, urbanicity was found to be positively correlated with an individual’s PRS_D_. In another longitudinal UKB study (n = 490780), participants with a high PRS_D_ had a 15% higher risk of developing MDD during a median follow-up of 8.8 years when exposed to high levels of air pollution, with evidence for both additive (RERI up to 0.15) and multiplicative GxE interaction effects.

Regarding SES during adulthood, data from the ELSA study revealed a weak, multiplicative interaction between an individual’s PRS_D_ and level of wealth for baseline depressive symptoms (β up to 0.08), but not for trajectories of depressive symptom change during a 14-year follow-up period [34]. Here, a 1-SD increase in PRS_D_ was associated with an increase in the number of symptoms by 0.08 points in n = 6202 older adults with low wealth.

To conclude, data from large cohort studies suggest significant, yet small, interactions of an individual’s PRS_D_ with specific environmental exposures in adulthood, particularly air pollution (RERI up to 0.15), outdoor light exposure (HR up to 1.05), and socioeconomic status (β up to 0.08) [34, 61, 62]. In contrast, for other exposures such as healthy lifestyle factors, physical activity, night shift work, and music engagement, only additive effects were reported [58–60, 63].

#### Cumulative lifetime adversity

We further retrieved studies investigating the interplay of PRS_D_ and cumulative lifetime adversity on depressive phenotypes. Drawing on 9599 participants of the CONVERGE study, Peterson et al. [66] reported no interaction between an individual’s PRS_D_ and self-reported lifetime trauma/SLE on MDD risk. Notably however, a GWAS stratified by lifetime trauma/SLE within the CONVERGE sample identified three new loci in participants with no history of adversity, where significant GxE effects were observed. In the largest study to date, Coleman et al. [64] contrasted genetic influences on MDD stratified by self-reported lifetime trauma exposure in 35269 MDD patients and 63451 controls from the UKB. Consistent with the stress-diathesis hypothesis, the authors observed a small additive interaction of PRS_D_ and lifetime trauma on MDD risk (β = 0.011), that was further reflected in a greater SNP-based heritability of MDD in trauma-exposed (24%) compared to non-exposed (12%) individuals. Subsequent simulation studies indicated that this effect was not confounded by a strong, positive rGE between PRS_D_ and lifetime trauma observed in this sample. A subsequent UKB study (n = 102182) conducted GxE analyses at the level of individual symptoms to account for the large, clinical heterogeneity observed in MDD patients [69]. Here, self-reported lifetime trauma and PRS_D_ were associated with specific patterns of current depressive symptoms and a positive, additive GxE interaction emerged for six out of nine current depressive symptoms (b up to 0.026), as well as for current (b = 0.158) and lifetime MDD risk (b = 0.015). The authors concluded that respective interactions between PRS_D_ and lifetime trauma in the UKB cohort are largely driven by overall current depression and not symptom-specific factors. Respective findings were partly confirmed by a longitudinal study comprising 18532 MDD patients and 20184 controls from the iPSYCH2012 cohort [68]. In this study, risk of early-onset depression was found to increase depending on an individual’s PRS_D_ and self-reported lifetime trauma/SLE load, with evidence for significant GxE interactions on the multiplicative (HR = 0.96) and additive (RERI = 0.09) scales. However, the effect sizes were small and in opposite directions. In contrast, no such GxE interaction between PRS_D_ and self-reported lifetime trauma on MDD risk was observed in a recent study on 1389 military service Veterans [70].

In summary, large cohort studies ( ~ 100000 participants) provide evidence for significant interactions of PRS_D_ and lifetime trauma on current and lifetime MDD as well as depressive symptoms, consistent with the stress-diathesis model [64, 69]. Similar findings of small but inconsistent GxE effects were reported in iPSYCH [68], while smaller studies found no evidence [66, 70]. Reported effect sizes were consistently small (β up to 0.158; b up to 0.026; HR = 0.96; RERI = 0.09), and some associations may be partly confounded by rGE [64].

### Studies investigating the interaction between PRS_D_ and environmental exposures on depression-related intermediate phenotypes

Beyond clinical outcomes, several studies have examined PRS_D_ x environment interactions (PRS_D_xE) on intermediate phenotypes relevant to depression pathophysiology, including cognitive and neuropsychological functions, inflammatory and neuroendocrine markers, and brain imaging measures. Although these phenotypes are not specific to depression, they can provide valuable insights into potential pathways and mechanisms underlying GxE effects. Overall, evidence for PRS_D_xE interactions across the intermediate phenotypes investigated is limited and inconsistent. While some studies report significant associations such as effects on neonatal brain structure, prefrontal activation under social stress, brain network connectivity, chronic inflammation, and cognitive biases, most large-scale investigations have not found supporting evidence. A detailed overview of these studies is provided in Supplement 3, both in narrative form and as a summary table.

## Discussion

Here, we provide a systematic review of studies that have taken a polygenic risk score approach to study GxE interaction on depressive phenotypes. Respective studies investigated a broad range of adverse and protective environmental exposures across the lifespan with a particular focus on trauma, SLEs, social environments and (un)healthy lifestyle factors. While the majority of individual studies reported significant main effects of an individual’s PRS_D_ and different environmental influences on depressive phenotypes, the overall evidence for PRS_D_xE interactions was considerably heterogeneous (as illustrated in a Sankey plot, Supplement 6). Findings of significant PRS_D_xE interactions mostly stem from large cohort studies, in particular, when recent environmental and lifetime exposures were considered. Among studies reporting significant GxE effects (29/69), the target sample size had a median of 5224 with an interquartile range (IQR) of 37534 and the corresponding discovery GWAS sample size had a median of 479299 (IQR = 650754). In studies without significant interaction effects, the target samples tended to be smaller (median = 3009, IQR = 7647), as were the discovery GWAS samples (median = 459014, IQR = 453815). These nominal differences should be interpreted with caution given the heterogeneity of environmental exposures and study designs.

Two general conclusions can be drawn from this review. First, PRS_D_xE interactions, if at all, add a small amount of explained variance in depressive phenotypes to the corresponding additive model and may thus require large samples to be reliably detected. Bearing in mind that studies summarized in this review rely on PRS_D_ that at best explained 3.2% of the variance in depression, even moderately-sized samples may lack sufficient power to detect GxE interactions of small effect size. Notably, some of the largest cohort studies on PRS_D_xE to date indeed revealed significant interactions between an individual’s PRS_D_ and CT as well as adult trauma/SLE (n = 38945, [31]), lifetime trauma (n > 100000, [64, 69]) and more specific exposures during adulthood such as time spent in outdoor light (n = 380976, [61]) and air pollution (n = 490780, [62]) on depression. Moreover, data from the UKB indicated that the SNP-based heritability of MDD was twice as large in trauma-exposed as compared to non-exposed individuals (24% vs 12%, [64]). Besides differences in statistical power, another crucial source of heterogeneity between studies derives from different strategies on how to select relevant SNPs for PRS_D_ calculation, ranging from very restrictive to more liberal *p*-value thresholds. Importantly, most studies that conducted sensitivity analyses across multiple *p*-value thresholds, found GxE interaction effects to be highly dependent on the specific threshold used for PRS_D_ construction (e.g., [36]), highlighting the need to establish a clear consensus. An apparently reasonable strategy applied by most studies was to analyze GxE effects with a PRS_D_ based on the specific threshold that provided highest accuracy for predicting depressive outcomes (e.g., [49, 55]). However, while significant main effects (of PRS_D_) are generally proposed as desirable when testing for GxE [89], there is also evidence that this approach may miss key interaction effects. Intriguingly, a first GWEIS across 25 environments on neuroticism, a trait with strong genetic and phenotypic overlap with depression, suggested that none of the interacting SNPs, genes or gene sets showed any evidence of significant main effects [90].

Second, in a considerable number of studies summarized in this review and beyond [91, 92], different environmental exposures were found to depend on an individual’s PRS_D_, indicating significant rGE, with small effect sizes across different metrics ( |r| = 0.058–0.21, |b| = 0.49, |β| = 0.04–0.163, OR = 1.07–1.76, R² = 0.40–0.45% and HR = 1.09). The assumption that genetic predispositions influence an individual’s environment via passive, evocative and active mechanisms is not new [71], but has received new support from GWAS indicating that traditional environmental measures such as self-reported CT are confounded by substantial heritable components [93, 94]. For example, a high PRS_D_ could inflate the perception and reporting of adverse environments or may even increase the individual’s risk of exposing themselves [71]. As detailed elsewhere, rGE thus constitutes a potential source of spurious GxE interactions [73] and has been found to inflate type I errors in simulation studies, albeit to a very modest extent [27]. Some studies pursued this problem by incorporating SLEs that are expected to be less dependent on the participant’s own behavior in GxE analyses, which were also found to be less heritable in some (e.g., [44, 95]), but not all [92] studies. To minimize rGE due to perception biases, studies may further rely on multi-informant or more objective assessments of environmental exposures, where heritability estimates are suggested to be slightly lower (29% for self-report vs. 26% for informant report [72]). As a general conclusion, reporting of rGE should be mandatory in future GxE studies in order to evaluate the extent of potential confounding.

A limitation of this review refers to the substantial heterogeneity of individual studies regarding sample size, study design, PRS_D_ calculation (e.g., threshold used), as well as the type and timing of environmental exposures investigated, which hampers an integrative conclusion or meta-analytical approach. For example, cross-sectional studies relying on self-reported exposures are susceptible to mood-congruent recall bias, as depressive states may inflate the reporting of environmental adversity [96, 97]. In addition, depression outcomes were defined in markedly different ways: Lifetime history of depression or MDD status, usually assessed in case–control designs, differs fundamentally from dimensional measures of acute depressive symptoms that reflect short-term severity and may include subclinical cases. Such variation in conceptualization and measurement further hinders direct comparison of results across studies. Moreover, several of the included studies used partially overlapping samples and data sources (e.g., UK Biobank, PGC-derived datasets), with genetic and phenotypic information from the same individuals appearing in more than one study. While these studies often investigated different environmental exposures, such overlap reduces the independence of findings and complicates the interpretation of apparent replication.

While evidence for robust interactions of polygenic liability and environmental exposures on depressive phenotypes remains inconclusive, it is reasonable to assume that PRS_D_ are only beginning to reach their full potential in terms of predictive accuracy. The estimated SNP heritability and phenotypic variance explained by PRS_D_ is steadily rising proportional to the sample size of the GWAS they are built upon [15], thereby increasing predictive power to stratify individuals at risk upon specific environmental exposures. This upward trend is expected to continue by integrating genetic risk variants that are not yet captured by traditional PRS_D_ once whole-genome sequencing data from MDD patients become increasingly available [98]. Since sufficiently predictive PRS_D_ are a key prerequisite for well-powered GxE analyses, further improvements in score construction are expected to enhance the feasibility and precision of such studies. Besides their potential in aiding risk stratification, pathway-specific PRS based on neuroimaging, proteomic or other multi-omic data might further advance our etiological understanding of underlying biological mechanisms implicated in the interplay of GxE in MDD. In the past years, substantial progress has been made in constructing polygenic scores based on gene transcription profiles targeting specific biological systems implicated in MDD, including inflammation [99], cellular stress responses [100] and neurotransmitter signaling pathways [101, 102] that could be easily combined with measures of environmental exposures in future GxE studies (e.g., [103]). To conclude, recent advances in PRSs construction have the potential to improve the investigation of GxE effects in the pathogenesis of MDD, which may in turn inform more targeted prevention strategies for individuals at elevated risk.

## Supplementary information

Supplement
