# Supplementary material for: The predictive value of polygenic risk scores for depression in gene-environment interaction studies: a systematic review
Source: Transl Psychiatry. 2026 Feb 25;16:121. doi: 10.1038/s41398-025-03793-7 (PMC12960946; doi:10.1038/s41398-025-03793-7)
Supplement: Supplementary file 1 — Supplement [file 41398_2025_3793_MOESM1_ESM.docx]

# Supplement

# The predictive value of polygenic risk scores for depression in gene-environment interaction studies: a systematic review

**Supplement 1: Genome-wide association studies of depression phenotypes**

Supplementary Table 1 provides an overview of prior genome-wide association studies (GWAS) on depression that served as a basis for PRS_D_ calculation for those studies included in the current review. Respective GWAS were based on different depression phenotypes, including lifetime MDD, depressive symptoms or alternative methods to measure MD and, with one exception [1], exclusively included samples of European ancestry. Sample sizes ranged from 76,237 to > 1 million individuals. Variance explained by PRS_D_, if reported, varied between 0.5-3.2% and increased with larger sample sizes of respective GWAS.

One of the few GxE studies in a non-European sample included in the current review [2] based their PRS calculation on seven psychopathology-related PRS, including a PRS for „depression“, „ever depressed“ and „recent depression“, that were found to maintain an average of 72.2% predictive ability in individuals of Middle Eastern ancestry compared to those of European UK Biobank participants (referred to as UKB-portability-2022 [3]).

# In addition to the traditional single-trait GWAS, two studies included in our review [4,5] referred to multi-trait analyses of GWAS (MTAG), a method for joint analysis of summary statistics from GWAS of different, overlapping traits. Chen et al. [4] extracted SNPs for PRS_D_ construction that were associated with depressive symptoms only in a MTAG study on different depression phenotypes, neuroticism, and subjective well-being based on data from the UK Biobank, the Genetic Epidemiology Research on Adult Health and Aging and 23andMe (referred to as MTAG-2018 [6]). In another study by Kosciuszko et al. [5], PRS_D_ were calculated using summary statistics for single-trait depressive symptoms and based on a MTAG approach including depressive symptoms, subjective well-being, neuroticism, loneliness, and self-rated health derived from the Polygenic Index Repository (referred to as PIR-2021 [7]). Moreover, one study [8] used only summary statistics from the GERA sample (GERA-2018 [9]).

**Supplementary Table 1: Overview of GWAS on depression that served as the basis for PRS_D_ calculation in the studies reviewed**

| Author | GWAS | sample | n (ancestry) | cases/controls | number of SNPs analyzed | depression phenotype | main results |
| --- | --- | --- | --- | --- | --- | --- | --- |
| Ripke et al, 2013 [10] | PGC-MDD- 2013 | full sample  discovery sample:  PGC-MDD (9 independent samples)  replication sample:  7 independent cohorts | 76,237  (European)  18,759  57,478 | 16,023 MDD  60,214 controls  9,240 MDD  9,519 controls  6,783 MDD  50,695 controls | 1.2 million  554 SNPs with p < 0.001 from discovery analysis | MDD  lifetime MDD based on  structured diagnostic interview/clinician-administered checklist (DSM-IV criteria)  lifetime MDD based on  structured diagnostic interview/clinician-administered checklist (DSM-III/IV or ICD10 criteria) | overall results:   - no genome-wide sign. hit |
| Okbay et al., 2016 [11] | SSGAC-2016 | discovery sample:  PGC-MDD  UKB  GERA  replication sample:  23andMe | 180,866  (European)  368,890  (European) | 9,240 MDD 9,519 controls  -  7,231 MDD 49,137 controls  - | 1.2 million  11.2 million  17.1 million  54 SNPs with p < 10^-5^ from discovery analysis | spectrum of depressive phenotypes  lifetime MDD  (see above)  self-report of  depressive symptoms  lifetime MDD based on electronic medical records  (ICD-9 criteria)  MD based on self-reported clinical diagnosis or treatment of MDD | overall results:   - 2 sign. associated SNPs (replicated) - estimated SNP heritability: 4.7% - PRS_D_ variance explained: ~0.5% |
| Hyde et al., 2016 [12] | PGC- 23andMe-2016 | full sample  discovery sample:  23andMe  joint analysis of 23andMe  & PGC-MDD discovery sample  replication sample  independent 23andMe | 785,594  (European)  307,354  (European)  326,113  (European)  152,127  (European) | 206,227 MD/MDD  579,367 controls  75,607 MD  231,747 controls  84,847 MD/MDD  241,266 controls  45,773 MD  106,354 controls | 15 million  1.2 million  SNPs with p < 1.0×10^−5^ from joint analysis | spectrum of depressive phenotypes  lifetime MD (see above)  lifetime MDD/MD  (see above)  lifetime MDD/MD  (see above) | - 15 sign. associated loci (17 SNPs) - 2 sign. associated loci (5 additional at p < 5×10−8) - 1 sign. associated lead SNP, not replicated at p < 5×10−6 - estimated SNP heritability: 5.9-6.9% - 3 out of 5 sign. associated SNPs from discovery sample replicated |
| Wray et al., 2018 [13] | PGC-MDD-2018 | full sample  PGC-MDD2 (29 independent samples)  6 additional cohorts (including e.g. UKB, 23andMe) | 480,359  (European)  42,455  437,904 | 135,458 MDD/MD  344,901 controls  16,823 MDD  25,632 controls  118,635 MD 319,269 controls | 9.6 million | spectrum of depressive phenotypes  lifetime MDD  (see above)  MDD/MD based on structured  diagnostic interviews, electronic medical records, self-reports | overall results:   - 44 sign. associated loci - estimated SNP heritability: 8.7% - PRS_D_ variance explained: 1.9% |
| Howard et al., 2019 [14] | PGC-MDD & 23andMe 2019 | discovery sample:  PGC-MDD2  UKB  23andMe  replication sample:  independent 23andMe sample | 807,553 (European)    1,306,354  (European) | 246,363 MD 561,190 controls  43,204 MDD  95,680 controls  127,552 MD 233,763 controls  75,607 MD 231,747 controls  414,055 MD  892,299 controls | 8 million  SNPs with p < 5 ×10^−8^ discovery analysis | spectrum of depressive phenotypes  lifetime MDD  (see above)  MD based on self-reported clinical diagnosis or treatment of MDD  see above  see above | - 102 sign. associated SNPs, 269 genes, 15 gene sets - estimated SNP heritability: 8.9% - PRS_D_ variance explained: 1.5-3.2% - 87 of the 102 sign. associated SNPs replicated |
| Cai et al., 2020 [15] | UKB- 2020 | UKB | 332,629 (European) | depending on MD/MDD definition | 5.3 million | compared minimal (e.g., help-seeking*)* and strict (e.g., CIDI-based) definitions of MD/MDD based on self-report | - highest number of sign. associated loci (27) with “help-seeking” definition of depression - estimated SNP heritability: 11-32%^a^ - PRS_D_ variance explained: NR |
| Levey et al., 2021 [1] | MD-Meta  2021 | MD META (MVP + 23andMe + UKB/PGC + FinnGen)  Self-report depression META  (MVP +  23andMe + UKB/PGC +  FinnGen)  PHQ-2 meta  (MVP + UKB)  MVP African ancestry  replication sample MD META:  independent 23andMe sample | 1,154,267  (European)  1,114,383  (European)  286,821  (European)  59,600  (African American) | 340,591 MD  813,676 controls  312,009 MD  802,374 controls  25,843 MD  33,757 controls  455,350 MD  887,428 controls | NR  211 sign. associated SNPs from MD META | ICD code definitions of MD (e.g., diagnostic interviews, electronic medical records)  MD diagnosis based on self-reported depression  depressive symptoms (PHQ-2)  ICD code, self-reported MD, depressive symptoms (s.o.)  ICD code, self-reported MD (s.o.) | - MD META: 223 sign. associated SNPs, 178 loci - replication:   68% of associated SNPs sign.   - estimated SNP heritability: 5.5-11.3% - genetic correlation among different MD phenotypes: 0.71 to 0.84 - trans-ancestral MD META: 223 sign. associated SNPs, 183 loci |

*Note*. The term ‘MDD’ was used when referring to directly evaluated individuals meeting standard diagnostic criteria for major depressive disorder, whereas ‘MD’ was used when case status was determined by alternative methods;

*Abbreviations*. CIDISF: World Health Organization Composite International Diagnostic Interview Short Form; GERA: Genetic Epidemiology Research on Adult Health and Aging; HRS: Health and Retirement Study; MVP: Million Veteran Program; NR: not reported; SNP: single nucleotide polymorphism; PGC: Psychiatric Genomics Consortium; PHQ-2: Patient Health Questionnaire-2; UKB: UK Biobank

^a^ Lower SNP-based heritability observed with minimal compared to strict phenotyping.

**Supplement 2: Quality Assessment of included studies**

**Supplementary Table 2. Definitions of quality assessment criteria Q1–Q7**

| Code | Question |
| --- | --- |
| Q1 | Was a power calculation performed? |
| Q2 | Were the in- and exclusion criteria clearly described? |
| Q3 | Was there an external validation cohort? |
| Q4 | Was adjustment for multiple testing applied? |
| Q5 | Are the methods used to construct the PGS specified? |
| Q6 | What type of association analysis was used for the main comparison? |
| Q7 | Were confounders or covariates considered in the analyses? |

**Note:** Responses for Q1–Q5 & Q7 were coded as “yes” or “no” based on the information reported in each study. For Q6, the type of analysis employed is provided.

**Supplementary Table 3. Variable coding definitions**

| Variable | Code | Definition |
| --- | --- | --- |
| Study design | 1 | Prospective longitudinal (both exposure and outcome assessed prospectively over time) |
|  | 2 | Retrospective longitudinal (retrospective assessment of earlier exposures related to current or later outcomes) |
|  | 3 | Cross-sectional (exposure and outcome assessed at the same time) |
| Environmental factor assessment (E) | 1 | Objective measurement (e.g., Covid-19 pandemic, exam period) |
|  | 2 | Established interview |
|  | 3 | Register-based (e.g., clinical records, geographic information systems) |
|  | 4 | Established self-report questionnaire |
|  | 5 | Self-constructed measurement |
|  | B | Biomarker-based assessment (e.g., prenatal maternal vitamin D status) |
| Phenotype assessment (P) | 1 | not used (kept blank to maintain consistency with Environmental Assessment coding) |
|  | 2 | Established interview |
|  | 3 | Register-based (e.g., clinical records, geographic information systems) |
|  | 4 | Established self-report questionnaire |
|  | 5 | Self-constructed measurement |
|  | B | Biomarker-based assessment (e.g., (f)MRI, cortisol awakening response) |

**Notes:** Multiple codes in E and P (e.g., “4, 5”) = more than one assessment used in the study.

**Supplementary Table 4: Quality assessment of included studies**

| Autor | study design | study sample size (N) | discovery sample size (N) | assessment of ethnicity | pT | E | P | assessment of rGE | Q1 | Q2 | Q3 | Q4 | Q5 | Q6 | Q7 |
| --- | --- | --- | --- | --- | --- | --- | --- | --- | --- | --- | --- | --- | --- | --- | --- |
| Acosta, Kantojärvi, & Hashempouret al., 2020 | 1 | 105  (European) | 18,759 (European) | yes | <0.001, <0.05, <0.1, <0.2 | 4 | B | no | no | yes | no | yes | yes | standard multiple linear regression analyses & ANOVA | yes |
| Acosta, Kantojärvi, & Tuulari et al., 2020 | 1 | 105  (European) | 18,759 (European) | yes | <0.001, <0.05, <0.1, <0.2 | 4 | B | no | no | yes | no | yes | yes | standard multiple linear regression analyses & ANOVA | yes |
| Agerbo et al., 2021 | 2 | 35,680 (Danish) | 807,553 (European) | yes | 10 pTs tested (range: <5x10^-8^ to <1) | 3 | 3 | yes | no | yes | no | no | yes | Cox proportional hazard models for case-cohort designs | yes |
| Armitage et al., 2022 | 1 | 2,268 (European) | 807,553 (European) | yes | 11 pTs tested (range: <5x10^-8^ to <0.5) | 2 | 4 | yes | yes | yes | no | yes | yes | negative nominal regressions | yes |
| Arnau-Soler et al., 2019 | 3 | 4,919 (European) | 155,866 (European) | yes | 8 pTs tested (range: <5x10^-8^ to <=1) | 4 | 4 | yes | no | yes | no | yes | yes | mixed linear models | yes |
| Cao et al., 2021 | 1 | 339,767  (European) | 76,237 (European) | yes | none | 5 | 3 | no | no | yes | no | yes | yes | Cox proportional hazard regressions | yes |
| Chen et al., 2022 | 3 | 35,633 (European) | 332,629 (European) | yes | <5x10^-8^ | 4 | 5 | no | no | yes | no | no | yes | generalized linear regressions | yes |
| Chen & Pokhvisneva et al., 2024 | 1 | cohort 1 (European) 5,546  cohort 2 (European) 514 | 354,862 (European) | yes | range: p= 0.01–1.00 at intervals of 0.01 | 4 | 4 | yes | no | yes | yes^2^ | yes | yes | longitudinal models with generalized estimating equations (GEEs) | yes |
| Chen & Yang et al., 2024 | 2 | 247,828 (European) | 61,847 (European) | yes | none (PRS-CS) | 5 | 3, 5 | no | no | yes | no | no | yes | multivariable Cox proportional hazards regressions | yes |
| Choi, & Chen et al., 2020 | 1 | 3,079  (European) | 173,005 (European) | yes | 8 pTs tested (range: 5x10^-8^ to 1.0), best pT = 0.01 | 4, 5 | 4 | yes | no | yes | no | yes | yes | logistic regressions | yes |
| Choi, & Zheutlin et al., 2020 | 1 | 7,968 (European) | 500,199 (European) | yes | 10 pTs  tested  (range: 5x10^-^  ^8^ to 1.0; final  pT: 1.0)^1^ | 5 | 3 | yes | no | yes | no | yes | yes | logistic regressions | yes |
| Cleary et al., 2023 | 1 | 1,011 (European)  435  (European) | 807,553 (European) | yes | NR | 4 | 4 | no | no | yes | yes | no | yes | Zero-inflated Poisson regressions | yes |
| Coleman et al., 2020 | 3 | 98,720 (European) | 450,619  (European) | yes | 7 pTs tested (range: <0.001 to <0.5; final pT: 0.5)^1^ | 4 | 4 | yes | no | yes | no | yes | yes | linear & logistic regressions | yes |
| Colodro-Conde et al., 2018 | 3 | 5,221 (European) | 159,598 (European) | yes | 8 tested (range: <5x10^-8^ to p<1; final pT: p<1)^1^ | 4 | 4 | yes | no | yes | no | no | yes | linear mixed models | yes |
| Domingue et al., 2017 | 1 | 8,588  (non-Hispanic white) | 18,759 (European) & 180,866  (European) | yes | NR | 1^3^ | 4 | no | no | yes | no | no | yes | parametric nonlinear regressions | yes |
| Fang et al., 2020 | 1 | 5,227 (European) | 807,553 (European) | yes | 9 pTs tested (range: <5 ×10^−8^ to <0.5, main analyses: <1) | 1 | 4 | no | no | yes | yes  (for PRS_D_main effect) | no | yes | linear regressions, linear mixed models & logistic mixed models | yes |
| Fu et al., 2022 | 1 | 490,780 (European) | 807,553 (European) | yes | NR | 3 | 3 | no | no | yes | no | no | yes | Cox proportional hazard regression models | yes |
| Giannelis et al., 2021 | 3 | 52,078 (European) | 143,265 (European) | yes | 11 pTs tested (range: <5x10^-8^ to <1; final pT: 0.3)^1^ | 5 | 4 | no | no | yes | no | yes | yes | logistic regressions | yes |
| Goltermann et al., 2021 | 3 | 1,217 (European) | 480,359  (European) | yes | 1 | 4 | B | no | no | yes | no | yes | yes | General linear models | yes |
| Halldorsdottir et al., 2019 | study 1: 3  study 2: 1 | cohort 1: 466 (European)  cohort 2: 1,450 (European) | 480,359 (European) | yes | 7 pTs tested (range: <5x10^-8^ to <0.10; final pT: <0.05)^1^ | 4 | 2, 4  4 | no | yes | yes | yes | yes | yes | multivariate regressions | yes |
| Hayes et al., 2017 | 3 | 160 (European) | 76,237 (European) | yes | 6 pT tested (range: <0.05 to <0.50) | 2 | B | no | no | yes | no | yes | yes | linear regressions | yes |
| Iob et al., 2023 | 2 | 3,428 (British) | 807,553 (European) | yes | =1.0 | 2, 4 | 4  B | no | no | yes | no | no | yes | multinomial logistic regressions  (ordinal) logistic regression and ordinal logistic regressions | yes |
| Joo et al., 2022 | 3 | 5,853  (multi-ethnic) | 807,553 (European) | yes | =1.0 | 4 | 2 | no | no | yes | no | yes | yes | logistic regressions | yes |
| Kochunov et al., 2022 | 3 | 24,141 (Caucasian) | 138,884 (European) | yes | p = .05 | 4 | B | no | no | yes | no | yes | yes | linear regressions | yes |
| Kosciuszko et al., 2023 | 2 | 6,202 (British) | 1,306,090 (European) | yes | NR | 3, 5 | 4 | no | no | yes | no | no | yes | linear mixed-effects models (LMMs) with maximum likelihood estimation | yes |
| Lehto et al., 2020 | 3 | 243,480 (European) | 161,460 (European) | yes | 7 pTs tested (range: <5x10^-8^ to <0.5; final pT: <0.5)^1^ | 1^3^ | 5 | yes | no | yes | no | yes | yes | linear regression (continuous outcomes), multinomial logistic regression (categorical outcomes) & multivariate logistic regression models (rGE) | yes |
| Li et al., 2021 | 3 | 352 (Han-Chinese) | 480,359 (European) | yes | <5x10^-8^ | 3 | B | yes | no | yes | no | yes | yes | partial least square regression (PLSR) | yes |
| Lin et al., 2023 | 1 | 380,976 (European) | 61,847 (European) | yes | none  (PRS-CS) | 5 | 3 | no | no | yes | no | no | yes | Cox proportional hazards regression models | yes |
| Lipsky et al., 2023 | 3 | 1,389  (non-Hispanic white participants) | 480,359 (European) | yes | 1,001 pTs tested (range p= 0.0001 to 1); final pT: NR | 4 | 2 | no | no | yes | no | yes | yes | logistic regressions | yes |
| Misztal et al., 2023 | 3, 1 | 4,975 (European) | 807,553 (European) | yes | <0.05 | 4 | 4 | no | no | yes | no | yes | yes | linear & logistic regressions | yes |
| Mullins et al., 2016 | 3 | 2,669 (European)  512  (European) | 15,546 (European) | yes | 9 pTs tested (range: <0.0001 to <0.5) | 4  4 | 2 | yes | yes | yes | no  yes (only rGE) | yes | yes | logistic and linear regressions | yes |
| Musci et al., 2016 | 2 | 488 (mostly African American) | 18,759 (European) | yes | <0.05 | 4 | 4 | no | no | yes | no | no | yes | latent trait–state–occasion (LTSO) approach | yes |
| Musliner et al., 2015 | 3 | 8,761  (mostly European) | 18,759 (European) | yes | 10 pTs tested (range: <0.10 to <1.0; final pT: <0.4)^1^ | 5 | 4 | yes | no | yes | no | no | yes | logistic and negative binomial regression models | yes |
| Musliner et al., 2021 | 2 | 38,716 (European) | 807,553 (European) | yes | 1 | 3 | 3 | yes | no | yes | no | no | yes | Cox regressions | yes |
| Nelemans et al., 2021 | 1 | 327  (European) | 478,240 (European) | yes | <5x10^-8^; sensitivity analysis:  12 pTs tested (range: <5x10^-7^ to <0.5) | 4 | 4 | yes | no | yes | no | no | yes | Latent Growth Curve Models (LGCMs) | yes |
| Perret et al., 2023 | 1 | 748  (Canadian) | 807,553 (European) | yes | 4 pTs tested: (<0.01; <0.10; <0.50; <1.00) | 4 | 4 | yes | no | yes | no | no | yes | hierarchical linear regressions | yes |
| Peter et al., 2023 | 1 | 432  (European) | 807,553 (European) | no | ≤0.05, PRS-CS | 1 | 4, 5, B | no | no | yes | no | no | yes | two level linear mixed models | yes |
| Peterson et al., 2018 | 3 | 9,599  (Han Chinese) | 18,759 (European) | yes | <0.2 | 4 | 2 | yes | yes (post-hoc) | yes | no | yes | yes | logistic regressions | yes (only PCs) |
| Peyrot et al., 2014 | 3 | 1,985 (European) | 15,298 (European) | yes | 8 pTs tested (range: <0.001 to <0.5) | 2 | 2 | yes | no | yes | no | no | yes | logistic regression models | yes |
| Peyrot et al., 2018 | meta-analysis on mixed design studies | 5,765 (European) | 149,815 (European) | yes | 5 pTs tested (range: <0.01 to <1) | 4 | 2 | yes | yes | yes | no (meta-analysis) | no | yes | logistic regressions | yes |
| Pine et al., 2023 | 3 | 4,619 (European)  1,063 (African American) | 500,199 (European)  59,600 (African American) | yes | none (PRS-CS)  none (PRS-CSx) | 4, 2, 5 | B | yes | yes (for PRS_D_ main effect) | yes | no^4^ | yes | yes | linear mixed effects models | yes |
| Pitharouli et al., 2021 | 3 | 85,895 (European) | 143,265 (European) | yes | <0.3 | 4 | B | no | no | yes | no | yes | yes | linear regressions | yes |
| Qiu et al., 2017 | 1 | cohort 1 168 (Asian)  cohort 2: 85 (mostly Hispanic American) | 18,759 (European) | yes | <0.05, <0.1, <0.2 | 4 | B | no | no | yes | no^5^ | yes | yes | linear regressions | yes |
| Schür et al., 2019 | 1 | 516  (European) | 480,359 (European) | yes | 13 pTs tested (range:  <5x10^-8^ to <1) | 4 | 4 | yes | no | yes | no | yes | yes | paired two-tailed t-tests & linear regressions | yes |
| Smeeth et al., 2023 | 3 | 1,359  (Middle Eastern ancestry) | 437,669 (European) | yes | NR | 4 | 4 | no | no | yes | no | yes | yes | logistic regressions | yes |
| Steen et al., 2022 | 1 | 19,128  (mostly European) | 500,199 (European) | yes | <0.05 | 4 | 2, 4 | no | no | yes | no | yes | yes | generalized estimating equations (Poisson & logistic regression) | yes |
| Stringa et al., 2020 | 1 | cohort 1 (European): 590   cohort 2 (European): 491  cohort 3 (European): 631  cohort 4:  (European) 567 | 480,359 (European) | yes | <0.05 (main analysis) & <0.2 (sensitivity check) | 4 | 4 | yes | no | yes | no^6^ | no | yes | linear & logistic Generalized Estimating Equations (GEE) | yes |
| Sund et al., 2021 | 3 | 41,198 (Norwegian) | 807,553 (European) | yes | <5x10^-8^ | 3 | 4 | yes | no | yes | no | no | yes | Mixed effect logistic regression models & linear probability models | yes |
| Taylor et al. 2022 | 3 | 2,050 (British) | 807,553 (European) | yes | 1 | 2, 1 | 4 | no | no | yes | no | yes | yes | linear and logistic regression modelsMus | yes |
| Thorp et al., 2023 | 3 | 102,182 (European) | 430,775 (European) | yes | none (SBayesR) | 4 | 4 | no | no | yes | no | yes | yes | linear regressions & proportional odds logistic regression models | yes |
| Turner et al., 2023 | 1 | 331  (European) | 807,553 (European) | yes | =1, all SNPs used | 1 | 4 | no | no | yes | no | no | yes | random effects ANOVA & linear regressions | yes |
| Wang et al., 2020 | 1 | 2,938 (European) | 307,354 (European) | yes | <0.05 | B | 2 | no | yes | yes | no | no | yes | logistic regressions & linear trend tests | yes |
| Wang et al., 2023 | 3 | 38,945 (European)  2,865 (European) | 807,553 (European) | yes | 11 pTs tested (range:  <5x10^-8^ to <1) | 4 | 2  4 | yes | no | yes | no | yes | yes | linear mixed regressions | yes |
| Wesseldijk et al., 2023 | 3 | 5,648 (European) | 807,553 (European) | yes | NR | 5, 4 | 3, 4 | yes | no | yes | no | yes | yes | linear regressions | yes |
| Zavlis et al., 2024 | 1 | 377 (European) | 480,359 (European) | yes | 9 pTs tested (range: 0.001 to 1) | 4 | B, 4 | yes | yes | yes | no | yes | yes | linear mixed effects models | yes |
| Zhang et al., 2021 | 3 | 394 (Han-Chinese) | 480,359 (European) | yes | <5x10^-8^ | 5 | B | yes | no | yes | no^6^ | yes | yes | ROI-based fMRI analysis | yes |

*Notes.* Q1–Q7 definitions are provided in Supplementary Table 2; Study design, E, and P codes are defined in Supplementary Table 3.

*Abbreviations.* NR: not reported; PRS-CS: polygenic prediction method using continuous shrinkage (CS) priors; PRS-CSx: PRS-CS based on ancestry-matched discovery GWAS; pT: p-value threshold; rGE: gene-environment correlation; ROI-based fMRI analysis: functional magnetic-resonance imaging (fMRI) analysis restricted to predefined regions of interest (ROIs); SBayesR: Bayesian multiple regression method that jointly analyzes all SNPs while accounting for linkage disequilibrium between them.

^1^ final pT: p-value threshold which explained the highest variance and is therefore used in the main analysis;

^2^ only main effect for prenatal stress on symptoms;

^3^ interview-based assessment of an objective life event (e.g., death of spouse, adoption as a child)

^4^ analysed European and African Americans separately (two population strata);

^5^ two population strata;

^6^ analysed 4 cohorts separately and pooled;

^7^ internal split-sample validation.

**Supplement 3: Studies investigating the interaction between PRS_D_ and environmental exposures on depression-related intermediate phenotypes**

Beyond clinical outcomes, several studies have explored PRS_D_ x environment interactions on intermediate phenotypes relevant to depression pathophysiology, including cognitive and neuropsychological functions, inflammatory or neuroendocrine markers and brain imaging measures. While these phenotypes are not necessarily specific to depression, they may help elucidate potential pathways and mechanisms through which GxE patterns emerge.

***Brain imaging phenotypes***

Two large magnetic resonance imaging (MRI) studies specifically investigated PRS_D_ x childhood trauma (CT) interactions on depression-related brain phenotypes, but both yielded null results. In a first MRI study on 24,141 older adult participants from the UK Biobank (UKB), PRS_D_ and retrospectively reported CT were not found to interact on a brain vulnerability index that measured an individual's brain-wide similarity to expected MDD patterns based on previous meta-analyses [16]. Similarly, a cross-sectional analysis of MRI data from the Adolescent Brain Cognitive Development (ABCD) study, including 4,619 European and 1,063 African American children aged 9–10 years, revealed no evidence for a GxE interaction between PRS_D_ and CT (as well as parent income and an area deprivation index) on hippocampal volume [17].

Beyond CT-related exposures, a functional MRI (fMRI) study on 394 individuals found no evidence for an interaction between PRS_D_ and socio-economic status (SES) in either childhood or adulthood on depression-related brain function [18]. However**, a** significant GxE interaction between PRS_D_ and childhood urbanicity **emerged in the same cohort,** with higher PRS_D_ predicting greater suppression of medial prefrontal cortex engagement under social stress during a working-memory task, but only in participants raised in urban environments [18]. Extending this focus on environmental characteristics of the living context, Li et al. [19] examined a multi-ethnic sample of 352 healthy adults and found a significant interaction between PRSD and ambient air pollution on cortical network connectivity. More precisely, combined high exposures to fine particulate matter and relatively high PRS_D_ **(defined as > 0.5 SD above the mean)** disproportionately augmented working memory and stress-related effects on effective connectivity across cortical and subcortical brain networks. The authors further reported that under the combined condition of high PRS_D_ and high air pollution, the observed brain network connectivity patterns were spatially correlated with the expression patterns of depression-related genes in corresponding brain regions, as documented in the Allen Brain Atlas. Extending the scope beyond socio-environmental factors, Hayes et al. (2017) examined whether PRS_D_ interacts with traumatic brain injury to influence cortical thickness in a cross-sectional sample of 160 European war veterans, but found no evidence for such an interaction [20].

Finally, a series of studies has examined prenatal environmental exposures in relation to offspring brain phenotypes. Several MRI investigations assessed whether the child’s PRS_D_ **interacts with** prenatal maternal depression **on** structural brain characteristics relevant to MDD pathophysiology. In a sample of 168 mother-infant dyads from the Growing Up in Singapore Towards healthy Outcomes (GUSTO) study, the PRS_D_ was found to interact with maternal depressive symptoms on neonatal amygdala and hippocampal volumes and on shape and thickness of the orbitofrontal and ventromedial prefrontal cortex [21]. More precisely, the authors observed a positive association between cortical brain volume, shape and thickness and maternal depression in respective regions in those infants with higher PRS_D_ that remained significant after controlling for maternal PRS_D._ A somewhat contrary GxE interaction effect on amygdala and hippocampal volumes and shapes was reported for maternal SES, where negative associations were observed in neonates with high PRS_D_. In a replication cohort of 85 mother–infant dyads, the PRS_D_ was also found to interact with prenatal maternal depression on the infants’ amygdala volume, however, the direction of this effect was opposite to that observed in the discovery sample. Notably, both cohorts differed with regard to their ethnic background (Asian vs. mostly Hispanic American), indicating that effects of PRS_D_ might vary between different populations. In another MRI study by Acosta et al. [22] comprising 105 mother-infant dyads from the FinnBrain Birth Cohort Study, amygdala volumes were more positively related to maternal depressive symptoms (assessed at gestational wks 14, 24, 34) in infants with low compared to high PRS_D_ **(nominally significant for pT = 0.1 only, β = –0.66)_._** While the direction of this GxE effect paralleled the one observed in the Hispanic-American cohort of the study by Qiu et al. [21], it did not survive the correction for multiple comparisons or subsequent control analyses. Additional analyses indicated that GxE effects on brain volume were partly sex-specific (β = –14.52 for right hippocampal volume) and dependent on the specific gestational week at which maternal depression was assessed. Moreover, the authors reported no interaction of PRS_D_ and prenatal maternal depression on the infants’ dorsal striatal volumes [23].

***Inflammatory and neuroendocrine markers***

Three studies examined PRS_D_xenvironment interactions on peripheral biological markers. Regarding inflammation, one study found significant additive and multiplicative interactions between PRS_D_ and CT on C-reactive protein (largest OR = 1.05), a marker of low-grade systemic inflammation linked to MDD pathophysiology [24]. In contrast, a much larger UKB analysis (26,894 MDD cases, 59,001 controls) reported no evidence for such an interaction [25]. With regard to neuroendocrine markers, Peter et al. (2023) found no evidence for an interaction between PRSD and exam stress on cortisol awakening response in a 13-month longitudinal study of 218 law students under chronic academic stress and 214 controls [26].

***Cognitive and neuropsychological phenotypes***

Within the broader range of intermediate phenotypes, cognitive processes remain relatively unexplored in the context of PRS_D_xE interactions. A prospective longitudinal study on 337 adolescents (baseline mean age = 13.4 years) focused on the development of memory and interpretation biases that are implicated in the pathogenesis and maintenance of MDD [27]. Interestingly, the authors observed a significant interaction of an individual’s PRS_D_ and self-reported positive (but not negative) life experiences on cognitive outcomes (largest β = 0.11). In adolescents with high PRS_D,_ positive life experiences were associated with stronger positive interpretation biases for social situations, supporting the notion of vantage sensitivity (i.e., where PRS_D_ also confers sensitivity to positive environments).

Complementing these findings in a different cognitive domain, Goltermann et al. [28] examined neurocognitive performance in an adult sample of 547 MDD patients and 670 controls. In this cross-sectional analysis, PRS_D_ and retrospectively reported CT (neglect and abuse) showed no evidence of interaction on cognitive functioning.

In summary, evidence for PRS_D_ x environment interactions across the intermediate phenotypes investigated is limited and inconsistent. Although some studies have reported significant associations such as effects on neonatal brain structure, prefrontal activation under social stress, brain network connectivity, chronic inflammation and cognitive biases, most large-scale investigations have yielded null findings.

| Supplementary Table 5: Studies investigating the interaction between a PRS_D_ and environmental exposures on intermediate phenotypes | | | | | | | | | |
| --- | --- | --- | --- | --- | --- | --- | --- | --- | --- |
| study | **study design^1^** | **population (ancestry)** | **age (mean)** | **female (%)** | **PRS discovery sample** | **PRS p-value threshold (pT)** | **environmental exposure** | **predicted outcome** | **main results (effect size estimate [CI])** |
| Acosta, Kantojärvi, & Hashempouret al., 2020 | prospective longitudinal | 105 mother–infant dyads (European) | 26.1 days | 41.9 | PGC-MDD-2013 | <0.001, <0.05, <0.1, <0.2 | prenatal maternal depressive symptoms at wks 14, 24, 34 (EPDS) | amygdala & hippocampal volume  (MRI) | - nominally sign. interaction of PRS_D_ (for pT = 0.1 only) and prenatal maternal depression on right amygdala volume (β = −0.66) - sign. sex-specific GxE effect on right hippocampal volume (largest β = −1.70) - no sign. main effects of PRS_D_ and prenatal maternal depression (NR) |
| Acosta, Kantojärvi, & Tuulari et al., 2020 | prospective longitudinal | 105 mother–infant dyads (European) | 26.1 days | 41.9 | PGC-MDD-2013 | <0.001, <0.05, <0.1, <0.2 | prenatal maternal depressive symptoms  wks 14, 24, 34 (EPDS) | dorsal striatal volumes  (MRI) | - no sign. interaction of PRS_D_ and prenatal maternal depression on dorsal striatal volumes (NR) - sign. sex-specific main effect of PRS_D_ on caudate volumes (largest β = 97.9), main effect of prenatal maternal depression NR |
| Goltermann et al., 2021 | cross-sectional | 547 MDD  670 HC  (European) | 34.7 yrs | 62.4 | PGC-MDD-2018 | =1.0 | CT  (CTQ) | cognitive functioning  (neuro-cognitive test battery) | - no sign. interaction of PRS_D_ and CT on cognitive functioning (NR) - sign. main effects PRS_D_ (η²p = 0.021) and CT (η²p = 0.083) |
| Hayes et al., 2017 | cross-sectional | 160 war veterans  (European) | 31.4 yrs | 6.9 | PGC-MDD-2013 | 6 pT tested (range: <0.05 to <0.50) | traumatic brain injury  (BAT-L) | cortical thickness  (MRI) | - no sign. interaction of PRS_D_ and traumatic brain injury on cortical thickness (NR) - main effects NR |
| Iob et al., 2023 | retrospective longitudinal | 3,428 (British) | t1≈56.7 yrs (8 follow-ups over 14 yrs) | 55.4 | PGC-MDD & 23andMe 2019 | =1.0 | CT  (ELSA’s Life History interview), parental bonding (PBI) | chronic inflammation (CRP) | - sign. additive (NR) and multiplicative interaction of PRS_D_ and CT (largest OR = 1.05 [1.02, 1.09]) on chronic inflammation - sign. additive (NR) and multiplicative interaction of PRS_D_ and (low) parental bonding (OR = 1.01 [1.01, 1.02]) on chronic inflammation - sign. main effects of PRS_D_ (OR = 1.03 [1.01, 1.04]), CT (OR = 1.08 [1.07, 1.09]) and (low) parental bonding (OR = 1.04 [1.04, 1.05]) on chronic inflammation |
| Kochunov et al., 2022 | cross-sectional | 24,141 (Caucasian) | ≈63.3 yrs | 54.8 | PGC-MDD- 2019 (excluding 23andMe, PGC only) | <0.05 | CT  (CTS) | brain anatomic vulnerability index for MDD (MRI) | - no sign. interaction of PRS_D_ and CT on brain anatomic vulnerability index (NR) - no sign. main effect of PRS_D_ (NR), sign. main effect of CT (NR) - no sign. correlation of PRS_D_ and CT (NR) |
| Li et al., 2021 | retrospective longitudinal | 352 (multi-ethnic) | 24.4 yrs | 16.7 | PGC-MDD-2018 | <5x10^-8^ | air pollution within the last 6 months (air monitoring station) | brain network connectivity during a working memory task across stress contexts  (MRI) | - sign. interaction of PRS_D_ and air pollution on brain connectivity networks (NR) - sign. main effects of PRS_D_ (NR) and air pollution (NR) - no sign. correlation of PRS_D_ and air pollution (r = 0.005) |
| Peter et al., 2023 | prospective longitudinal | 218 stressed students  (European)  214 controls  (European) | 22.9 yrs  (6 assessments within 13 months  21.0 yrs  (6 assessments within 13 months) | 73.0  77.0 | PGC-MDD & 23andMe 2019 | ≤0.05 | exam period | cortisol awakening response | - no sign. interaction of PRS_D_ and exam stress (b = 21.59), no sign. 3-way interactions with time (b = 2.43) - no main effect of PRS_D_ and exam stress, but sign. interaction of exam stress x time on cortisol awaking response (b = −18.99) |
| Pine et al., 2023 | cross-sectional | 4,619 (European)  1,063  (African American) | 9-10 yrs  9-10 yrs | NR  NR | PGC-MDD & 23andMe 2019  MD-Meta  2021 (African American sample) | none  (PRS-CS)  none (PRS-CSx) | parent income  (self-report);  CT  (KSADS);  area deprivation  index | hippocampal volume  (MRI) | both samples:   - no sign. interaction of PRS_D_ and parent income, CT and area deprivation index on hippocampal volume (largest **β =**12.08**)** - no sign. main effect of PRS_D_ (largest β = 17.36) - no sign. main effects of parent income (largest β = −0.019), CT (largest β = −4.990) and area deprivation index (largest β = −0.487) - sign. correlation between PRS_D_ and deprivation (r = 0.07) |
| Pitharouli  et al., 2021 | cross-sectional | 85,895 (European)  26,894 MDD  59,001 HC | 56.0 yrs | 54.7 | PGC-MDD-2018 | <0.3 | CT  (CTS) | C-reactive protein  (CRP) | - no sign. interaction of PRS_D_ and CT on CRP levels (β = 0.003 [−0.0005, 0.006]) - sign. main effects of PRS_D_ (β = 0.017 [0.010, 0.024]) and CT (β = 0.034 [0.030, 0.037]) |
| Qiu et al., 2017 | prospective longitudinal | cohort 1 (Asian)  168 mother–infant dyads  cohort 2 (mostly Hispanic American)  85 mother–infant dyads | 40.1 wks  43.0 wks | 46.4  41.2 | PGC-MDD 2013 | <0.05, <0.1, <0.2 | prenatal maternal depressive symptoms and SES  (EPDS)  prenatal maternal depressive symptoms  (CES-D) | structural brain changes  (MRI) | - sign. interaction of PRS_D_ and prenatal maternal depressive symptoms on amygdala and hippocampal volumes and on shape and thickness of the orbitofrontal and ventromedial prefrontal cortex (NR) - sign. interaction of PRS_D_ and maternal SES on amygdala and hippocampal volumes and shapes (NR) - main effects of PRS_D_, prenatal maternal depressive symptoms and SES NR - sign. interaction of PRS_D_ and prenatal maternal depressive symptoms on amygdala volume (opposite direction to Asian sample) (NR) - main effects of PRS_D_ & prenatal maternal depressive NR |
| Zavlis et al., 2024 | prospective longitudinal | 377  (European) | t1=13.4 yrs  t2=14.6 yrs  t3=15.7 yrs | 51.0 | PGC-MDD-2018 | 9 pTs tested (range: 0.001 to 1) | positive and negative life experiences  (CASE) | memory bias (SRET);  interpretation bias  (AIBQ) | - sign. interaction of PRS_D_ and positive (but not negative) life experiences on memory/interpretation bias (largest β = 0.11 [0.05, 0.17]) - no main effect of PRS_D_ (largest β = −0.11 [−0.22, −0.01]), sign. main effects of negative/positive life experiences (largest β = 0.13 [0.08, 0.18]) - no sign. correlation between PRS_D_ and positive/negative life experiences (largest r = 0.09) |
| Zhang et al., 2021 | cross-sectional | 394  (Han Chinese) | ≈24.4 yrs | 51.0 | PGC-MDD-2018 | <5x10^-8^ | childhood urbanicity; childhood SES (self-report)  adult SES  (self-reported) | prefrontal cortex activity during a working memory task across stress contexts (fMRI) | - sign. interaction of PRS_D_ and childhood urbanicity on prefrontal brain functioning (NR) - no sign. interaction of PRS_D_ and childhood SES on prefrontal brain functioning (NR) - main effects of PRS_D_ and urbanicity NR - no sign. correlation of PRS_D_ and urbanicity - no sign. interaction between PRS_D_ and adulthood SES on prefrontal brain functioning (NR) - main effects of PRS_D_ and adulthood SES NR |

*Abbreviations*. AIBQ: The Adolescent Interpretation and Belief Questionnaire; b: unstandardized regression coefficient; BAT-L: Boston Assessment of TBI-Lifetime (TBI: traumatic brain injury); CASE: Child Adolescent Survey of Experiences; CES-D: Center for Epidemiologic Studies Depression Scale; CI: confidence interval; CRP: C-reactive protein; CT: childhood trauma; CTQ: Childhood Trauma Questionnaire; CTS: Childhood Trauma Screener; EPDS: Edinburgh Postnatal Depression Scale; fMRI: functional magnetic resonance imaging; HC: healthy controls; KSADS: Kiddie Schedule for Affective Disorders and Schizophrenia; MDD: major depressive disorder; MRI: magnetic resonance imaging; NR: not reported; OR = odds ratio; PRS-CS: polygenic prediction method using continuous shrinkage (CS) priors; PRS-CSx: PRS-CS based on ancestry-matched discovery GWAS; PRS_D_: polygenic risk score for depression; pT: p-value threshold; r = correlation coefficient; SES: socio-economic status; SRET: Self-Referential Encoding Task; sign.: significant; t: timepoint; wks: weeks; yrs: years; β: standardized regression coefficient; η²p = partial eta squared.

^1^ A study was classified as “cross-sectional” when exposure and outcome were assessed at the same time point in the same sample, “retrospective longitudinal” when the exposure was measured before the outcome in time, but both were obtained from past records and analyzed after outcome occurrence, or as “prospective longitudinal” when both environmental exposures and depressive outcomes were prospectively assessed.

**Supplement 6: Sankey plots**

Supplementary Figure 1 illustrates the distribution and flow of study characteristics across key dimensions: type and time of environmental exposure, discovery GWAS sample size used for PRSD calculation, depression-related outcome (symptoms or diagnosis), and whether a significant GxE effect was reported. The plots thereby complement the main tables by providing a visual overview of how heterogeneous exposures, genetic discovery power, and outcome definitions are represented across the included studies.

**
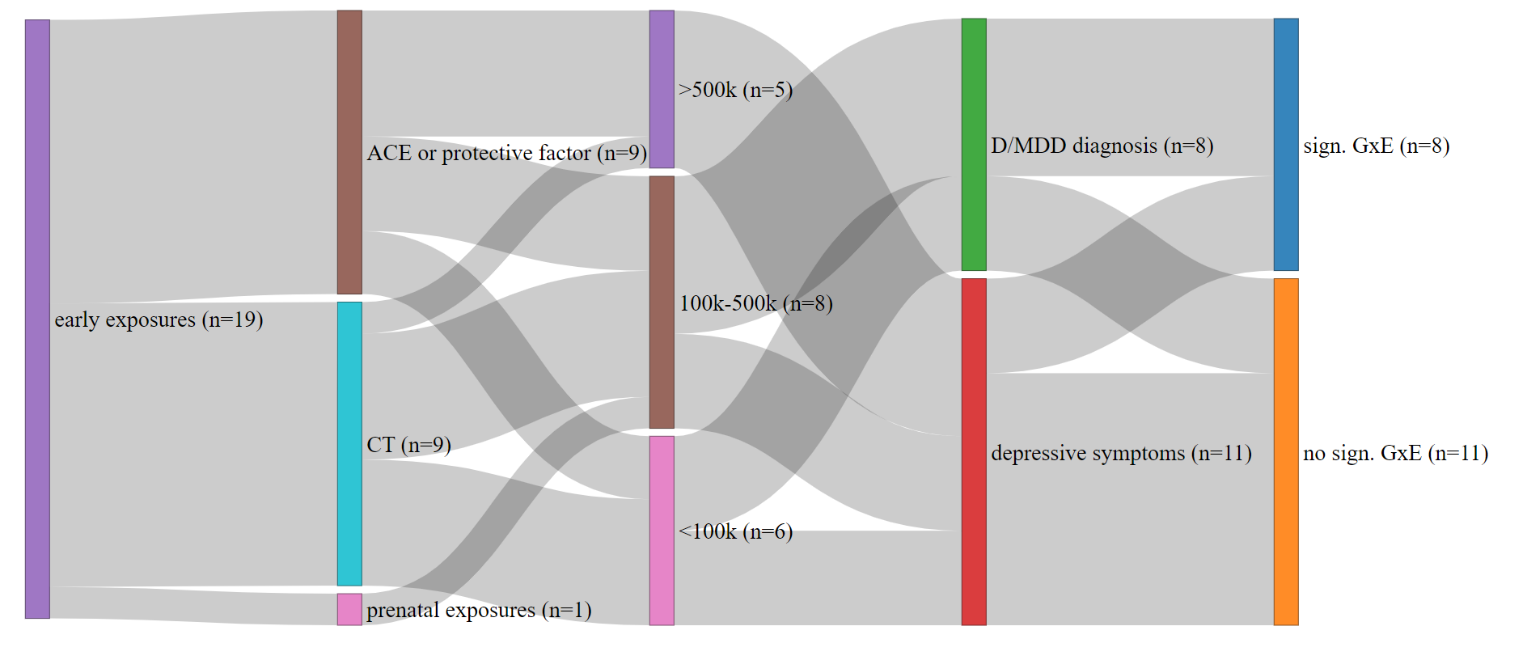
***
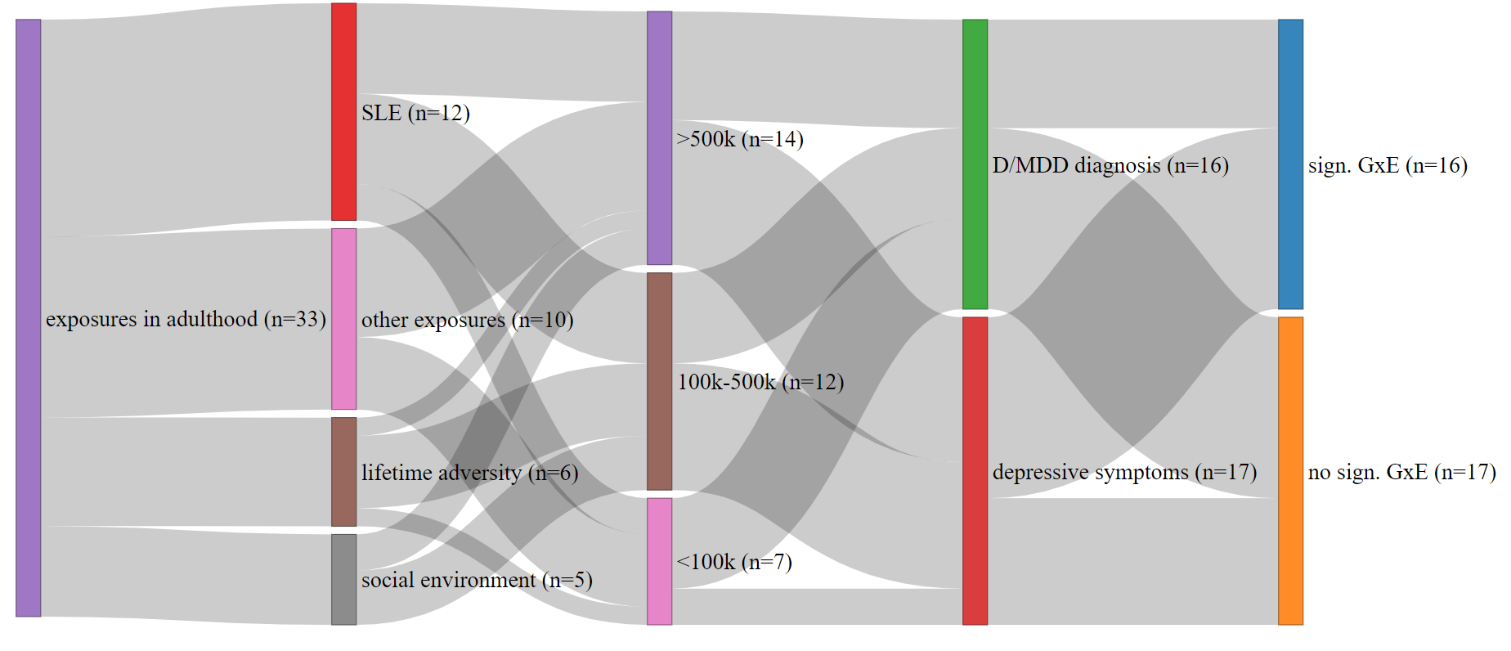
***Supplementary Figure 1: Sankey plots of included studies on gene–environment (GxE) interactions with early-life and adulthood environmental exposures on depressive symptoms or depression/MDD diagnosis.**

**B**

**Timing of exposure Type of exposure GWAS sample size Phenotype GxE interaction effect**

**A**

*Note.* n = number of studies. Timing of exposure refers to the life period during which participants experienced the environmental exposure. Type of exposure: ACE = adverse childhood experience; CT = childhood trauma; SLE = stressful life experiences; lifetime adversity = cumulative adversity across the lifespan. GWAS sample size indicates the size of the genome-wide association study (GWAS) used to calculate the polygenic risk score for depression (expressed in thousands, k). Phenotype = measured outcome (D/MDD diagnosis = depression or major depressive disorder diagnosis). GxE interaction = whether the study reported a significant (sign.) gene–environment interaction effect. A Studies investigating early-life environmental exposures. B Studies investigating environmental exposures.
Click the link “Supplementary Figure 1” below to view the interactive plot.

**Interactive Plots:** [Supplementary Figure 1](https://osf.io/zf7ja/files)

**References**

1. Levey DF, Stein MB, Wendt FR, Pathak GA, Zhou H, Aslan M, et al. Bi-ancestral depression GWAS in the Million Veteran Program and meta-analysis in >1.2 million individuals highlight new therapeutic directions. Nat. Neurosci. 2021;24:954–63.

2. Smeeth D, May AK, Karam EG, Rieder MJ, Elzagallaai AA, Van Uum S, et al. Risk and resilience in Syrian refugee children: A multisystem analysis. Dev. Psychopathol. 2023;35:2275–87.

3. Privé F, Aschard H, Carmi S, Folkersen L, Hoggart C, O’Reilly PF, et al. Portability of 245 polygenic scores when derived from the UK Biobank and applied to 9 ancestry groups from the same cohort. Am. J. Hum. Genet. 2022;109:12–23.

4. Chen LM, Pokhvisneva I, Lahti-Pulkkinen M, Kvist T, Baldwin JR, Parent C, et al. Independent Prediction of Child Psychiatric Symptoms by Maternal Mental Health and Child Polygenic Risk Scores. J. Am. Acad. Child Adolesc. Psychiatry 2024;63:640–51.

5. Kosciuszko M, Steptoe A, Ajnakina O. Genetic propensity, socioeconomic status, and trajectories of depression over a course of 14 years in older adults. Transl. Psychiatry 2023;13:68.

6. Turley P, Walters RK, Maghzian O, Okbay A, Lee JJ, Fontana MA, et al. Multi-trait analysis of genome-wide association summary statistics using MTAG. Nat. Genet. 2018;50:229–37.

7. Becker J, Burik CAP, Goldman G, Wang N, Jayashankar H, Bennett M, et al. Resource profile and user guide of the Polygenic Index Repository. Nat. Hum. Behav. 2021;5:1744–58.

8. Chen Y, Yang H, Zhang Y, Zhou L, Lin J, Wang Y. Night shift work, genetic risk, and the risk of depression: A prospective cohort study. J. Affect. Disord. 2024;354:735–42.

9. Zhu Z, Zheng Z, Zhang F, Wu Y, Trzaskowski M, Maier R, et al. Causal associations between risk factors and common diseases inferred from GWAS summary data. Nat. Commun. 2018;9:224.

10. Ripke S, Wray NR, Lewis CM, Hamilton SP, Weissman MM, Breen G, et al. A mega-analysis of genome-wide association studies for major depressive disorder. Mol. Psychiatry 2013;18:497–511.

11. Okbay A, Baselmans BML, De Neve JE, Turley P, Nivard MG, Fontana MA, et al. Genetic variants associated with subjective well-being, depressive symptoms, and neuroticism identified through genome-wide analyses. Nat. Genet. 2016;48:624–33.

12. Hyde CL, Nagle MW, Tian C, Chen X, Paciga SA, Wendland JR, et al. Identification of 15 genetic loci associated with risk of major depression in individuals of European descent. Nat. Genet. 2016;48:1031–6.

13. Wray NR, Ripke S, Mattheisen M, Trzaskowski M, Byrne EM, Abdellaoui A, et al. Genome-wide association analyses identify 44 risk variants and refine the genetic architecture of major depression. Nat. Genet. 2018;50:668–81.

14. Howard DM, Adams MJ, Clarke TK, Hafferty JD, Gibson J, Shirali M, et al. Genome-wide meta-analysis of depression identifies 102 independent variants and highlights the importance of the prefrontal brain regions. Nat. Neurosci. 2019;22:343–52.

15. Cai N, Revez JA, Adams MJ, Andlauer TFM, Breen G, Byrne EM, et al. Minimal phenotyping yields genome-wide association signals of low specificity for major depression. Nat. Genet. 2020;52:437–47.

16. Kochunov P, Ma Y, Hatch KS, Gao S, Jahanshad N, Thompson PM, et al. Brain‐wide versus genome‐wide vulnerability biomarkers for severe mental illnesses. Hum. Brain Mapp. 2022;43:4970–83.

17. Pine JG, Paul SE, Johnson E, Bogdan R, Kandala S, Barch DM. Polygenic Risk for Schizophrenia, Major Depression, and Post-traumatic Stress Disorder and Hippocampal Subregion Volumes in Middle Childhood. Behav. Genet. 2023;53:279–91.

18. Zhang X, Yan H, Yu H, Zhao X, Shah S, Dong Z, et al. Childhood urbanicity interacts with polygenic risk for depression to affect stress-related medial prefrontal function. Transl. Psychiatry 2021;11:522.

19. Li Z, Yan H, Zhang X, Shah S, Yang G, Chen Q, et al. Air pollution interacts with genetic risk to influence cortical networks implicated in depression. Proc. Natl. Acad. Sci. 2021;118:e2109310118.

20. Hayes JP, Logue MW, Sadeh N, Spielberg JM, Verfaellie M, Hayes SM, et al. Mild traumatic brain injury is associated with reduced cortical thickness in those at risk for Alzheimer’s disease. Brain J. Neurol. 2017;140:813–25.

21. Qiu A, Shen M, Buss C, Chong YS, Kwek K, Saw SM, et al. Effects of Antenatal Maternal Depressive Symptoms and Socio-Economic Status on Neonatal Brain Development are Modulated by Genetic Risk. Cereb. Cortex 2017;27:3080–92.

22. Acosta H, Kantojärvi K, Tuulari JJ, Lewis JD, Hashempour N, Scheinin NM, et al. Sex-specific association between infant caudate volumes and a polygenic risk score for major depressive disorder. J. Neurosci. Res. 2020;98:2529–40.

23. Acosta H, Kantojärvi K, Hashempour N, Pelto J, Scheinin NM, Lehtola SJ, et al. Partial Support for an Interaction Between a Polygenic Risk Score for Major Depressive Disorder and Prenatal Maternal Depressive Symptoms on Infant Right Amygdalar Volumes. Cereb. Cortex 2020;30:6121–34.

24. Iob E, Ajnakina O, Steptoe A. The interactive association of adverse childhood experiences and polygenic susceptibility with depressive symptoms and chronic inflammation in older adults: a prospective cohort study. Psychol. Med. 2023;53:1426–36.

25. Pitharouli MC, Hagenaars SP, Glanville KP, Coleman JRI, Hotopf M, Lewis CM, et al. Elevated C-reactive protein in patients with depression, independent of genetic, health, and psychosocial factors: Results from the UK Biobank. Am. J. Psychiatry 2021;178:522–9.

26. Peter HL, Giglberger M, Streit F, Frank J, Kreuzpointner L, Rietschel M, et al. Association of polygenic scores for depression and neuroticism with perceived stress in daily life during a long‐lasting stress period. Genes Brain Behav. 2023;22:e12872.

27. Zavlis O, Parsons S, Fox E, Booth C, Songco A, Vincent JP. The effects of life experiences and polygenic risk for depression on the development of positive and negative cognitive biases across adolescence: The CogBIAS hypothesis. Dev. Psychopathol. 2024;1–10.

28. Goltermann J, Redlich R, Grotegerd D, Dohm K, Leehr EJ, Böhnlein J, et al. Childhood maltreatment and cognitive functioning: the role of depression, parental education, and polygenic predisposition. Neuropsychopharmacology 2021;46:891–9.
